# Characterization of early psychosis patients carrying a genetic vulnerability to redox dysregulation: a computational analysis of mechanism-based gene expression profile in fibroblasts

**DOI:** 10.1038/s41380-023-02034-x

**Published:** 2023-03-31

**Authors:** Basilio Giangreco, Daniella Dwir, Paul Klauser, Raoul Jenni, Philippe Golay, Martine Cleusix, Philipp S. Baumann, Michel Cuénod, Philippe Conus, Nicolas Toni, Kim Q. Do

**Affiliations:** 1 Center for Psychiatric Neuroscience, Department of Psychiatry, Lausanne University Hospital and University of Lausanne (CHUV-UNIL), Lausanne, Switzerland; 2 Service of Child and Adolescent Psychiatry, Department of Psychiatry, Lausanne University Hospital and University of Lausanne (CHUV-UNIL), Lausanne, Switzerland; 3 Service of Community Psychiatry, Department of Psychiatry, Lausanne University Hospital and University of Lausanne (CHUV-UNIL), Lausanne, Switzerland; 4 Service of General Psychiatry, Department of Psychiatry, Lausanne University Hospital and University of Lausanne (CHUV-UNIL), Lausanne, Switzerland

**Keywords:** Neuroscience, Diagnostic markers, Predictive markers

## Abstract

In view of its heterogeneity, schizophrenia needs new diagnostic tools based on mechanistic biomarkers that would allow early detection. Complex interaction between genetic and environmental risk factors may lead to NMDAR hypofunction, inflammation and redox dysregulation, all converging on oxidative stress. Using computational analysis, the expression of 76 genes linked to these systems, known to be abnormally regulated in schizophrenia, was studied in skin-fibroblasts from early psychosis patients and age-matched controls (*N* = 30), under additional pro-oxidant challenge to mimic environmental stress. To evaluate the contribution of a genetic risk related to redox dysregulation, we investigated the GAG trinucleotide polymorphism in the key glutathione (GSH) synthesizing enzyme, glutamate-cysteine-ligase-catalytic-subunit (*gclc*) gene, known to be associated with the disease. Patients and controls showed different gene expression profiles that were modulated by GAG-*gclc* genotypes in combination with oxidative challenge. In GAG-*gclc* low-risk genotype patients, a global gene expression dysregulation was observed, especially in the antioxidant system, potentially induced by other risks. Both controls and patients with GAG-*gclc* high-risk genotype (*gclc*GAG-HR) showed similar gene expression profiles. However, under oxidative challenge, a boosting of other antioxidant defense, including the master regulator Nrf2 and TRX systems was observed only in *gclc*GAG-HR controls, suggesting a protective compensation against the genetic GSH dysregulation. Moreover, RAGE (redox/inflammation interaction) and AGMAT (arginine pathway) were increased in the *gclc*GAG-HR patients, suggesting some additional risk factors interacting with this genotype. Finally, the use of a machine-learning approach allowed discriminating patients and controls with an accuracy up to 100%, paving the way towards early detection of schizophrenia.

## Introduction

Schizophrenia (SZ) is triggered by a first psychotic episode which often follows a period of several months during which patients may suffer from poorly specific symptoms. Among young people with an at-risk mental state (ARMS), only a small proportion will transit to full-blown psychosis. Therefore, a better identification of those at true risk of developing a first psychotic episode is crucial to set up early intervention strategies. In this context, there is a need for novel mechanism-based biomarkers that would allow a better understanding of the pathophysiology and improve identification of ARMS young people.

Intensive research from the last decades suggests that the etiopathogeny of SZ involves both environmental and genetic risk factors, interacting early during brain development. Many environmental factors, that were shown to increase the risk for SZ [1–3] may induce oxidative stress and inflammation, leading to brain maturation impairments [4–6]. Moreover, GWAS data point to various polymorphisms, that confer higher risk for SZ, in immune, antioxidant and NMDAR related genes [7, 8]. Therefore, one hypothesis of interest is that these genetic and environmental-induced risk factors lead to NMDAR hypofunction, redox dysregulation and inflammation [9–18], all converging on excessive oxidative stress, which affects, among others, the maturation of GABAergic parvalbumin expressing interneurons (PVI) [4, 19], critical for cognition.

Among the genetic risk associated with SZ, a polymorphism in the gene of the key synthetizing enzyme for the major non-enzymatic antioxidant glutathione (GSH), the glutamate-cysteine ligase-catalytic subunit (*gclc*), was found to be associated with the disease, conferring a genetic vulnerability to redox imbalance [9, 10]. Individuals bearing a high number of the GAG trinucleotide repeat in the *gclc* gene (“High-risk” genotype) showed decreased GSH level in the prefrontal cortex (PFCx) [11] as well as decreased GCL activity and protein level after an additional oxidative challenge in their fibroblasts [9]. Furthermore, a metabolomic study revealed abnormal regulation of the GSH related metabolites after an additional oxidative challenge, as well as increased oxidative-induced lipid damage in fibroblasts of early psychosis (EP) patients carrying the “High-risk” genotype [12].

Here, we used a computational analysis approach to investigate the differences of SZ-related gene expression profile in fibroblasts from EP patients and age-matched controls, with the GAG-*gclc* genetic vulnerability to redox dysregulation and an additional pro-oxidant challenge, that stimulates the antioxidant defenses [13, 14] and mimics an additional environmental challenge that may interact with the genetic vulnerability background. Our aim was to identify (1) the role of a genetic background of vulnerability (GAG-*gclc* polymorphism) to redox dysregulation on different pathways in patients, (2) pathways that may be altered by other risk factors (genetic or environmental) in patients and (3) potential protective pathways that may be induced in controls bearing the same genetic vulnerability to redox dysregulation. Overall, this pathway analysis may lead to a specific biological profile of gene expression that would discriminate between patients and controls.

## Material and methods

### Subjects recruitments

EP patients were recruited from the Treatment and Early Intervention in Psychosis Program (TIPP), a 3-year specialized program for patients aged between 15 and 35 years old who met the threshold criteria for psychosis according to the Comprehensive Assessment of At-Risk Mental States criteria) [20, 21]. More information is presented in the Supplementary.

### Fibroblasts culture and treatment

Fibroblasts from skins biopsies of EP patients and age-matched healthy controls were prepared as previously described [9, 12]. In order to minimize heterogeneity in our samples, only males were included in this study. Culture of fibroblasts from the 4 groups (*N* = 15), namely GAG-*gclc* low-risk (LR) controls, GAG-*gclc* high-risk (HR) controls, GAG-*gclc* LR patients and GAG-*gclc* HR patients, were cultured in parallel until their 5^th^ cell passage and then treated for 18 h either with tert-butylhydroquinone (tBHQ) at 50 µM, to induce an oxidative stress, or with vehicle alone (dimethyl sulfoxide (DMSO), 0.05% final). After treatment, cells were harvested with trypsin for 3 min, collected for centrifugation (10 min at 1000 × *g*) and washed with PBS. For RNA extraction, cells were frozen as pellet. For GCL activity, cells were frozen in 1 ml of PBS.

### RNA extraction

Total RNA was extracted form fibroblast pellet with the NucleoSpin RNA kit (Macherey-Nagel). RNA quality and integrity were evaluated with the RIN method (Agilent RNA 6000 Nano Kit) such that all samples have a RIN numbers greater than 8.

### Gene expression Fluidigm

Gene expression was measured with the Pair Delta Gene assays and reagents with EvaGreen dye using a Fluidigm BioMark Genetic Analysis Platform at Georgia Institute of Technology, Atlanta, USA. Gene expression was normalized to 6 housekeeping genes (Supplementary Table 1).

### GCL activity

GCL activity was performed with an in-house method as described previously [9]. Briefly, a fluorescence-based microtiter plate assay was used to measure GCL activity, determined as the difference between GSH synthesis in unblocked and buthionine sulfoximine (BSO)-blocked wells per minute and per milligram of protein. Samples of interest are analyzed in the presence of a master-mix containing 400 mM Tris pH8, 40 mM ATP, 20 mM L-glutamic acid, 2 mM EDTA, 20 mM sodium borate, 2 mM serine, 40 mM MgCl2, with or without BSO (15 mM). The reaction starts when 2 mM cysteine is added to the wells and incubated for 45 min at 37 °C. The reaction is stopped with 5-sulfosalicylic acid (200 mM) and proteins are precipitated to isolate the GSH. The level of GSH is measured with the addition of 10 mM Naphthalene-2,3-Dicarboxaldehyde (NDA) that yields a fluorescent signal in contact of thiols.

### Computational analysis

Statistical and computational analysis were performed using R studio, JMP and Matlab software. The sample size was chosen according to our previous study conducted on metabolomic analyses on fibroblasts of the same cohort [12]. Data were tested for normality of distribution and homogeneity of variance with the Shapiro-Wilk Test and Bartlett test respectively (with acceptance value of *p* > 0.05 for both). Then, a two-way-ANOVA analysis with 3 factors was used to reveal group (patient or control), genotype (GAG-*gclc* HR and LR) or treatment (tBHQ and DMSO) effect for each gene expression, corrected for multiple comparison. A PCA, followed by a factorial analysis with a parsimax rotation, and a multivariate correlation matrix, with multiple correction, were estimated. To facilitate the interpretation of components coming from the initial PCA analysis, we relied upon using a rotation procedure. Among other rotation methods, the Parsimax criteria [22] is targeted toward a simple structure that serves as a proxy to facilitate a biological interpretation of the two axes. A discriminant analysis was done using the 2 or 4 groups, followed by the Support Vector Machine (SVM) algorithm. More details are described in the Supplement.

## Results

**Fig. 1:**
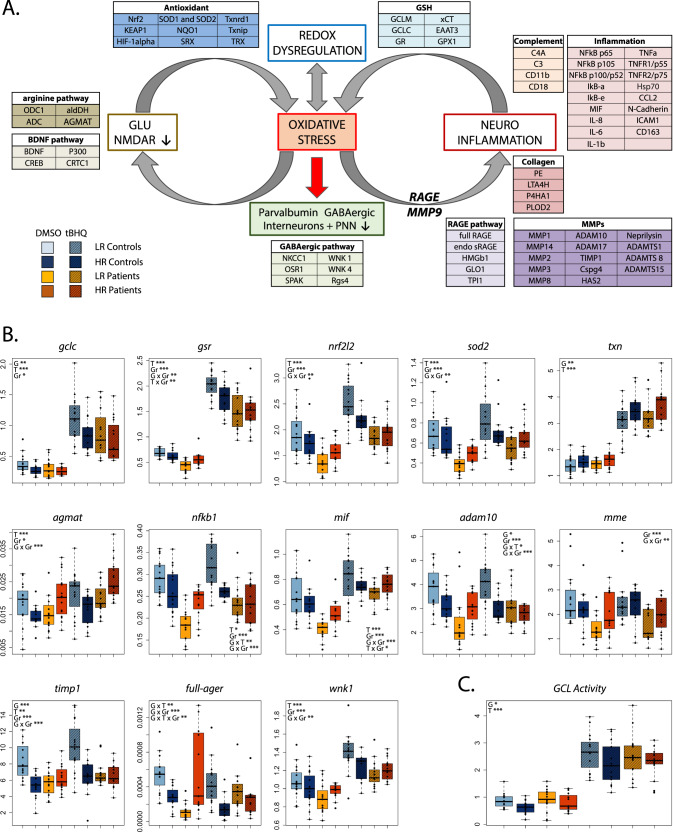
General scheme of hypothesis-driven gene selection in different pathways and the corresponding boxplots of selected gene expression. **A** Genes selected based on the hypothesis that redox dysregulation, neuroinflammation and NMDAR hypofunction act in a feedforward loop of processes to converge on oxidative stress, which affects parvalbumin expressing interneurons during their maturation as a core pathophysiological mechanism in SZ. Genes that were shown to be altered in SZ, were selected in 10 different pathways, namely the antioxidant pathway, the GSH related pathway, inflammation, the complement, the collagen synthesis/degradation, MMPs, RAGE, arginine metabolism, BDNF and GABAergic maturation. The protein name is represented in this scheme and corresponding gene name is established in Supplementary Fig. 1. **B** Boxplot of selected genes, after a two-way-ANOVA analysis with 3 factors, the treatment (T: tBHQ and DMSO), the status (Gr: patient or control) and the genotype (G: GAG-*gclc* HR and LR). Genes from different pathways were found to be significantly different between patients and controls, and the GAG-*gclc* polymorphism was found to modulate the different responses in patients and in controls, which highlight the important role of the genetic background for redox vulnerability. **C** GCL activity was significantly decreased in the HR genotype patients and controls, highlighting the functional consequence of the GAG-*gclc* polymorphism. Data are expressed as mean ± s.e.d. (*N* = 15) *P < 0.05; **P < 0.01; ***P < 0.001.

Following the hypothesis of an interaction between the redox balance, inflammation and NMDAR inducing GABAergic interneurons impairments (Fig. 1A), we analyzed by fluidigm the expression of 76 genes related to these system (Fig. 1A; Supplementary Table 1), which were previously shown to be linked to SZ (Fig. 1A; Supplementary Table 1). In the redox system, *antioxidant defenses*, such as the GSH [11, 15] and the thioredoxine/sulfiredoxine (TRX/SRX) system [16] were investigated, as well as Nrf2, Keap1 and Hif1, the master transcription factors that regulate them [17, 18, 23, 24]. Regarding the *inflammatory pathways* [25, 26], the master transcription regulator NFkB/IkB and their downstream effectors, cytokines and cell-adhesion molecules [27–30] were chosen for the analysis but also genes related to the extracellular matrix modulation, such as *matrix metalloproteinases* (MMPs) [31, 32] and enzymes involved in *collagen formation/degradation. The complement system*, which was recently shown to be involved in SZ pathophysiology [33–35] was also added to this inflammatory pathway. For the interaction between the redox and the inflammatory systems, we also investigated some specific mechanism-related pathway such as the *receptor for advanced glycation end-product (RAGE)* that has been linked to GABAergic impairments in EP patients and induces a feedforward loop of oxidative stress and inflammation in an animal model of SZ [36]. The *arginine pathway* was also explored, as being involved in the nitric oxide formation, aldehyde detoxification but also in inflammation and NMDAR modulation [12, 37–39]. Finally, some genes related to the consequence of these pathways on GABAergic interneurons maturation were added to the analysis, such as *BDNF pathway* [40–42] and *NKCC1/KCC2 system*, which is involved in the early maturation of parvalbumin expressing interneurons [43–45].

### Patients display an altered antioxidant and inflammatory response that is modulated by the GAG-*gclc* polymorphism

Gene expression changes were analyzed using a three-way-ANOVA analysis with 3 factors, namely, the treatments (T: tBHQ vs. DMSO), the groups (Gr: patients vs. controls) and the genotypes (G: HR vs. LR).

For the antioxidant defenses pathway, as expected, tBHQ treatment induced a significant response, with mostly an upregulation (Fig. 1B) [9]. We confirmed the decreased *gclc* gene expression in the HR (Fig. 1B, Supplementary Fig. 1A) [9] under both DMSO and tBHQ conditions. Noteworthy, the *txn* gene expression was increased in the HR as compared to the LR, suggesting a compensation of this antioxidant system over the GSH system (Fig. 1B, Supplementary Fig. 1B). Overall, patients showed a significant decrease in the antioxidant genes expression at basal level but also after tBHQ treatment (Fig. 1B, Supplementary Fig. 1A, B).

Some inflammatory genes also responded to the tBHQ treatment (Fig. 1B, Supplementary Fig. 2A, B) as well as the matrix extracellular proteinases (MMP) and the collagen formation/degradation system (Fig. 1B, Supplementary Figs. 3A and  4C), showing the tight interaction between oxidative stress and inflammation [28]. Comparing between patients and controls, gene expressions were mostly decreased in patients, showing the alteration in inflammatory response and extracellular matrix regulation.

Finally, the BDNF and the NKCC1/KCC2 systems were regulated by the treatment and by the group effect (patient vs control) (Fig. 1B, Supplementary Fig. 4A, B).

For all pathways investigated, the GAG-*gclc* polymorphism modulated the different responses in both patients and in controls, highlighting the important role of this genetic background for redox vulnerability.

We further explored the antioxidant impairments by measuring GCL activity in the fibroblasts of patients/controls, HR/LR. GCL activity was significantly increased by the tBHQ treatment with a significant effect of the HR/LR genotype (Fig. 1C). As previously shown in fibroblasts of chronic SZ patients [9], GCL activity was decreased in the HR, highlighting the functional consequence of the GAG-*gclc* polymorphism (Fig. 1C).

### The gene expression profile of GAG-*gclc* HR controls is similar to patients

We used a PCA to highlight the biological pathways that contributed the most to the differences between the 8 groups (*N* = 15, total of 120 samples, 76 genes).

**Fig. 2:**
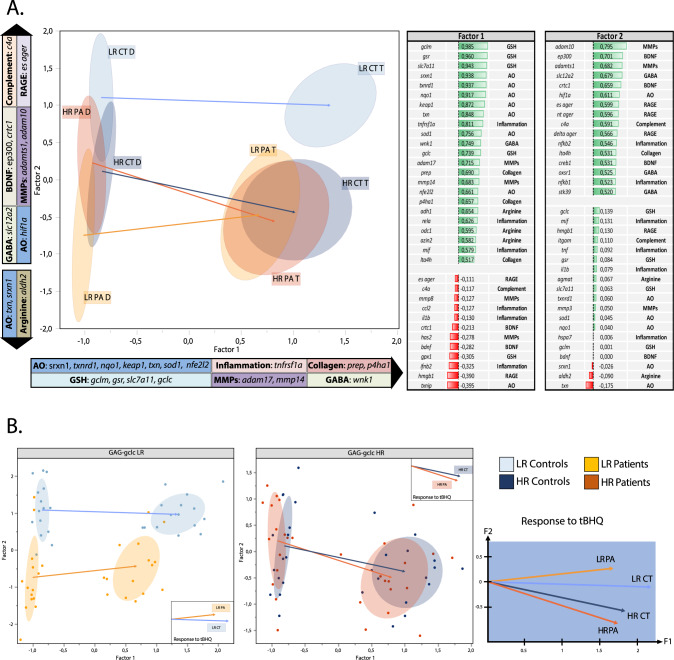
Factorial analysis with rotation to visualize the pathways that are differentially regulated in patients and controls. **A** Graphic representation of the 4 groups (*LR CT, HR CT, LR PA, HR PA*) under DMSO (*D*) and tBHQ (*T*), and a table of the genes of each pathway that were the highest contributors to the rotated factor1 and factor2. **B** Graphic representation of GAG-*gclc* LR and HR genotype separated, with their corresponding vectors of response to tBHQ, showing the similarities or differences between patients and controls with the same genotype (GAG-*gclc*).

PC1 and PC2 contributed for 27.2% and 12.2% of the variability (Supplementary Fig. 5A), but were not associated with biologically relevant differences. Therefore, to ensure a better interpretation, the data were rotated with the orthogonal parsimax criteria so that the obtained factor1 represented mostly the response to tBHQ treatment (Fig. 2A, Supplementary Fig 6A), and the factor2 represented the different groups of patients and controls, with the genotype (Fig. 2A, Supplementary Fig 6A). In the highest contributors to factor1 and factor2, we found one or two genes from each different pathway, that represented and separated the most the different groups (Fig. 2A, Supplementary Table 3, Supplementary Figs 6 and 7).

By looking first at the general position of each group treated with DMSO or tBHQ on the PCA analysis, we found that under DMSO condition, the LR controls and the LR patients were clearly separated, while the HR controls and the HR patients overlapped (Fig. 2A). The response to tBHQ showed different signatures for the LR and HR individuals (both patients and controls). The initial position under DMSO and the difference induced by tBHQ lead to well separated position after treatment in the both LR patients and controls (Fig. 2B, light orange and light blue arrows) while the HR controls and patients converge to an overlapping position (Fig. 2B, deep blue and red arrows). Of major interest, after tBHQ, the HR controls and the LR and HR patients overlapped completely, while the LR controls were completely separated from the other groups (Fig. 2A). Thus, cells from HR controls show multiple overlap with LR and HR patients under tBHQ and are different from cells from LR controls.

The factor1 and factor2 revealed the genes and related pathways that contributed most to the group separation, and more interestingly, their relative expression among groups. We found that tBHQ treatment induced an overall increase in the antioxidant defenses, which was more pronounced in the LR controls (Fig. 2A). Indeed, the HR individuals as well as the LR patients failed to increase Nrf2 (*nfe2l2*) and GSH related genes (*gclm*, *gsr* and *slc7a11*) at the same level as the LR controls, but increased the TRX/SRX system (*txn* and *srxn1*), suggesting a compensatory mechanism. The inflammatory pathway (*tnfrsf1a*, *rela*) and collagen degradation/formation (*prep*, *p4ha1*, *lta4h*) were increased in the LR controls after tBHQ stimulation, suggesting their contribution to the normal tBHQ response (Fig. 2A), which was less pronounced in the HR subjects and in the LR patients. Moreover, after tBHQ treatment, several MMPs (*adam17, adam10, adamts1* and *mmp14*) were differentially regulated in the LR controls compared to the other groups. Finally, the endogenous soluble form of RAGE (*es-ager*) was increased in the LR controls, compared to the other groups, after tBHQ stimulation, as a potential protective mechanism to prevent full RAGE gene expression (Fig. 2A)

Together, these results show that LR patients and LR controls are very distinct, probably reflecting the effect of risk factors not related to Gclc (Fig. 2B). In contrast, HR controls are very similar to patients, in particular after tBHQ treatment (Fig. 2B), suggesting that they engage compensatory mechanisms that prevent the development of the pathology.

### Different regulatory profile between patients and controls in interaction with the genetic risk for redox dysregulation

**Fig. 3:**
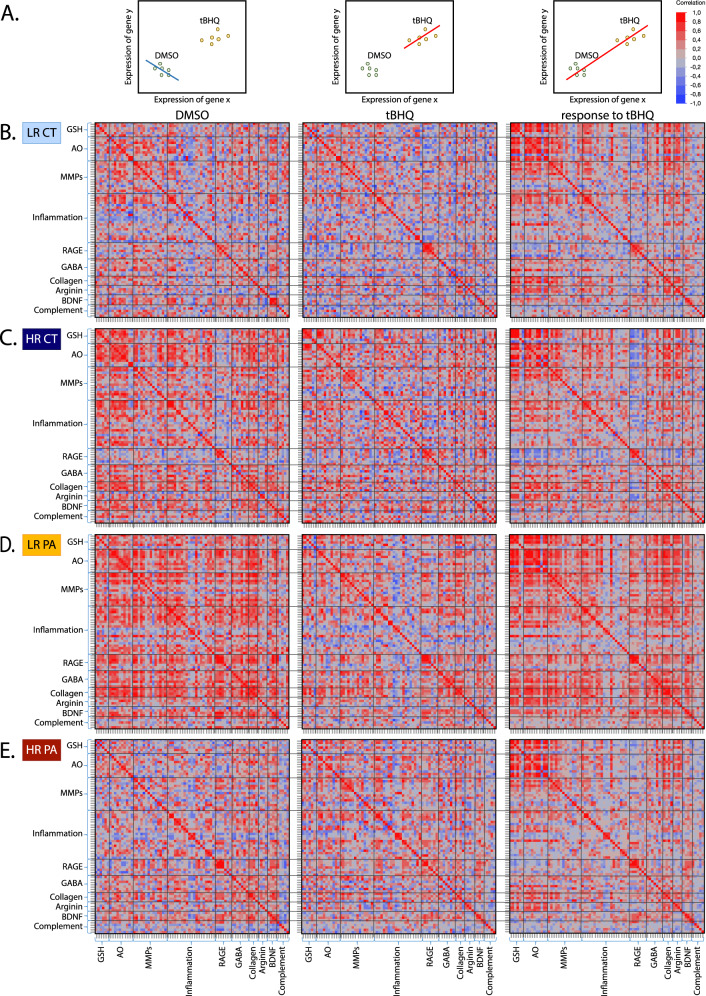
Correlation matrix with multiple correction to investigate the regulation of the various genes between pathways, under DMSO or tBHQ conditions, and in the response to tBHQ. **A** Schematic representation of the color meaning for DMSO or tBHQ conditions, and in the response to tBHQ. Each gene was correlated to all the others in the DMSO and tBHQ condition separately, highlighted by the blue color when the correlation is negative and red when it is positive. Two genes can be negatively correlated under DMSO condition, but positively correlated under tBHQ treatment. In order to evaluate the response to tBHQ, correlations between genes were also investigated by looking at the correlation of the values of DMSO and tBHQ on the same graph. When tBHQ induced an upregulation of the two genes, the correlation was positive (red), while the correlation was negative (blue) when tBHQ induced a downregulation. Correlation matrix for the 4 groups, namely **B** the GAG-gclc- LR controls (LR CT), **C** HR controls (HR CT), **D** LR patients (LR PA) and **E** HR patients (HR PA). All groups show a different correlation profile, suggesting some regulatory processes related to the GAG-*gclc* HR polymorphism in patients, other risk factors in LR patients, but also protective mechanism in the HR controls.

We investigated pathway regulation underlying the differences or similarities between patients and controls, by performing a multivariate analysis with a correlation matrix, assuming that correlation may reflect the regulation between different pathways. Each gene was correlated to all the others under the DMSO and tBHQ conditions separately or together (Fig. 3A).

In the LR controls, tBHQ treatment elicited a positive correlation in gene expression of the antioxidant systems (Fig. 3B), as expected. Interestingly, positive correlations were also found between the antioxidant system and the inflammatory systems, as well as between the antioxidant system and the collagen and arginine pathways (Fig. 3B), highlighting the important role of the redox balance in the regulation of these cellular processes.

The HR controls showed increased overall positive correlation under DMSO condition, as compared to LR controls, suggesting some regulatory compensatory processes taking place at basal level due to their genetic background (Fig. 3C). Among them, increased correlation between the antioxidant/GSH and the other pathways suggests a major role of the redox regulation. Of note, the RAGE pathway was negatively correlated with all other pathways in the response to tBHQ, a signature only found in the HR controls (Fig. 3C).

Compared to LR controls, the LR patients presented a completely different profile, showing a high increase in overall positive correlations under DMSO conditions, a slight increase in overall positive correlations under tBHQ, and a further increase in the positive correlation in the response to tBHQ (Fig. 3D). This profile being different between patients and controls, without the GAG-*gclc* polymorphism, implies other pathological processes independent of this specific genetic risk.

In contrast, the HR patients did not display correlation under DMSO conditions, nor an increase thereof after tBHQ treatment, despite their genetic vulnerability background that may lead to increased oxidative stress. This suggests an impaired response to oxidative challenge (Fig. 3E), potentially linked to the disease.

In general, every single group shows a particular correlation profile, suggesting the intervention of regulatory processes related to the GAG-*gclc* polymorphism in HR patients, to other risk factors in LR patients, or to protective mechanism in the HR controls.

### Discriminant analysis highlight pathways involved in HR controls protection and pathological conditions in patients

In order to highlight the pathways involved in the differences between all 4 groups, we first performed a discriminant analysis to compare two-by-two the different groups under DMSO and tBHQ conditions.

#### LR controls vs LR patients (Fig. 4A)

This first comparithe hypothesis of an interaction beould differentiate between patients and controls not carrying the GAG-*gclc* polymorphism. Although these groups are not genetically predisposed to a redox dysregulation linked to the GAG-*gclc* polymorphism, the antioxidant genes (*nfe2l2/keap1*, *gclm*, *gsr* and *sod2*) were nonetheless discriminative as they were decreased in patients, suggesting that risk factors other than the GAG-*gclc* polymorphism may induce a redox dysregulation in these patients. Moreover, NFkB (*nfkb)*, as well as some MMPs (*mme* and *timp1*) were also found to play a role in this segregation and were decreased in patients, emphasizing the contribution of inflammatory mechanisms.

**Fig. 4:**
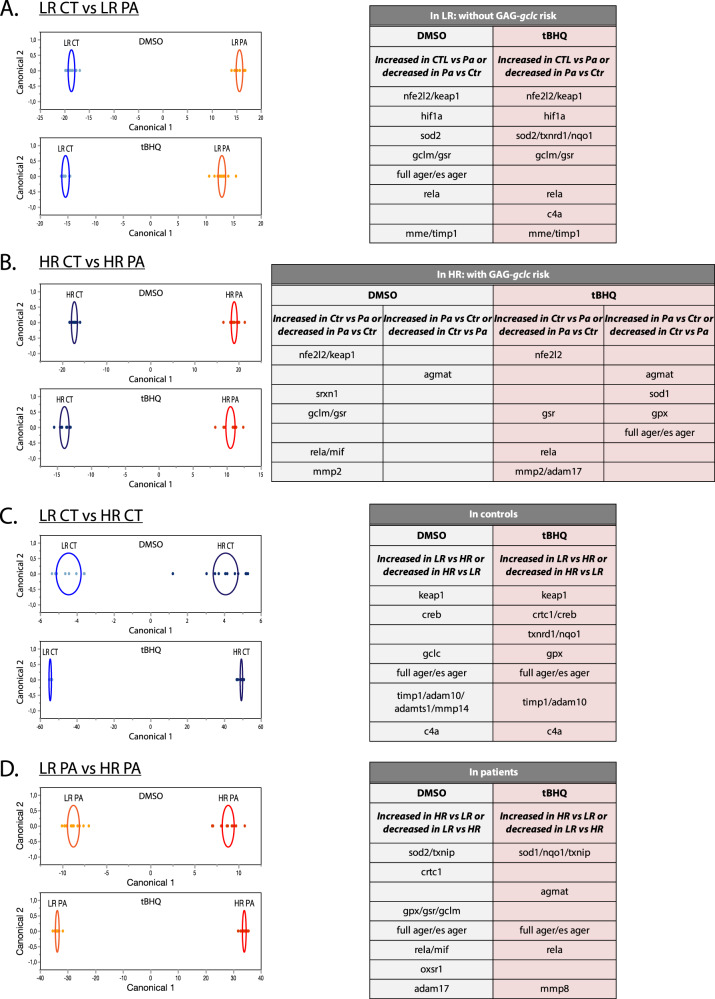
Discriminant analysis to compare two-by-two the different groups under DMSO and tBHQ conditions. **A** Discriminant analysis on GAG-*gclc* LR patients (LR PA) and controls (LR CT) to identify pathways that would differentiate between patients and controls, independently of the GAG-*gclc* risk. **B** Discriminant analysis on GAG-*gclc* HR patients (HR PA) and controls (HR CT) to identify potential protective pathways, but also highlight pathways induced by other risks in interaction with the GAG-*gclc* polymorphism that would lead to the disease. **C** Discriminant analysis on GAG-*gclc* LR (LR CT) and HR (HR CT) controls to investigate the potential protective mechanism in the controls bearing the GAG-*gclc* polymorphism. **D** Discriminant analysis on GAG-*gclc* LR (LR PA) and HR (HR PA) patients to point out the pathways related to the GAG-*gclc* risk, but also to reveal some pathways related to other risks, in order to stratify sub-groups of patients. Genes that are contributing the most to the discrimination are showed in the corresponding tables.

#### HR controls vs HR patients (Fig. 4B)

The next comparison gave an insight into potential protective pathways (present in controls, but not in patients), but also highlighted pathways induced by other risks in interaction with the GAG-*gclc* polymorphism that could lead to the disease condition (present in patients but not in controls). In both DMSO and tBHQ conditions, HR controls displayed increased antioxidant (*nfe2l2*, *srx*, *gclm*, *gsr*) and inflammatory (*nfkb*, *mif*) gene expression levels, as compared to HR patients. This suggests that despite the same genetic vulnerability towards redox dysregulation as HR patients, HR controls display antioxidant protection that differentiates them from the patients. Of interest, agmatinase (*agmat*) was increased in patients as compared to controls. Furthermore, the RAGE pathway (*full ager* and *es ager*) was increased in patients after tBHQ treatment.

#### LR controls vs HR controls (Fig. 4C)

Exploring the potential protective mechanism in the controls bearing the GAG-*gclc* polymorphism, we compared LR and HR controls. Among the genes that discriminated the most both groups, the antioxidant system was decreased in the HR controls as compared to LR controls, especially after the tBHQ treatment, in line with the genetic vulnerability to oxidative stress of HR controls. Moreover, the RAGE pathway (*full ager* and *es ager*) and some MMPs (*timp1*, *adam10*, *adamts1* and *mmp14*) also participated to this discrimination, suggesting their interaction with the GAG-*gclc* polymorphism.

#### LR patients vs HR patients (Fig. 4D)

The discriminant analysis on the LR and the HR patients was performed to point out the pathways related to the GAG-*gclc* risk, but also to reveal some pathways related to other risks, in order to stratify sub-groups of patients. The discrimination was mostly driven by the RAGE pathway and agmatinase (*agmat*), which were increased in the HR patients. This suggests a link between the GAG-*gclc* genetic risk and these specific genes.

Together, this discriminant analysis revealed novel pathways differentially expressed in patients and controls, linked to GAG-*gclc* polymorphism. In particular, in LR patients we found a redox dysregulation that was not related to the GAG-*gclc* polymorphism, suggesting a role of other risk factors. Moreover, HR controls displayed an effective compensatory antioxidant system, such as the TRX/SRX, potentially providing a protective mechanism. Noteworthy, our analysis revealed RAGE and AGMAT as underlying the pathological conditions, especially in HR patients.

### Machine-learning-based discrimination between patients and controls

A major challenge in psychiatry is the development of biomarkers that would help to identify individuals who would benefit the most from an early and specific intervention. In the context of SZ, it is crucial to improve the discrimination, among ARMS individuals, between those who will convert or not to psychosis. A first step in that direction is the evaluation of the power of our gene expression set to discriminate between EP patients and controls.

**Fig. 5:**
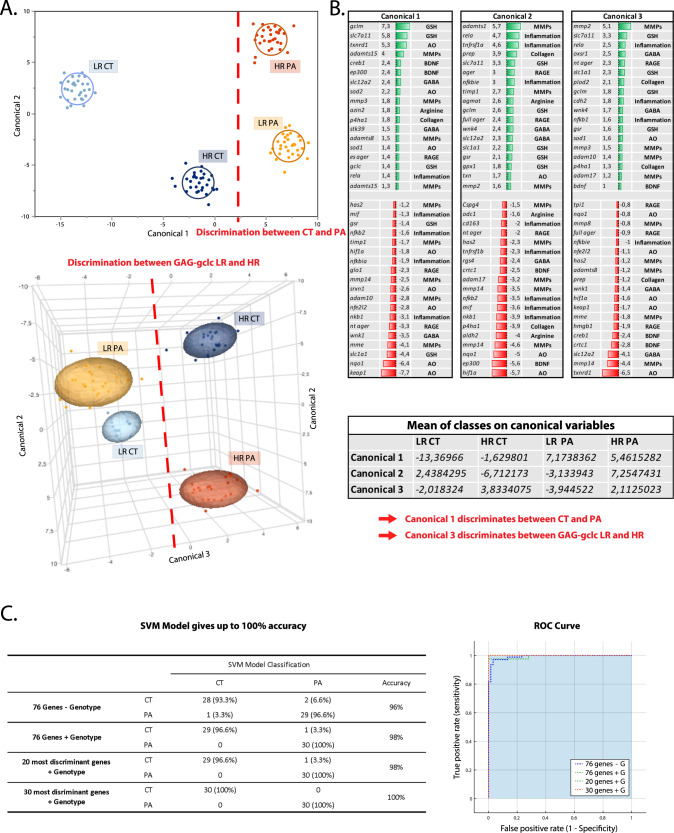
A discriminant analysis on all groups together found the best split of the data based on preselected groups and a machine-learning approach identified patients and controls. **A** Representation of the discriminant analysis between groups, in 2D and 3D. By entering into the analysis all the genes from all groups treated with DMSO or tBHQ, 3 canonical components were able to fully discriminate LR controls, HR controls, LR patients and HR patients. **B** The list of genes which composed the canonical 1, 2 and 3. The canonical 1 discriminates between patient and controls, while the canonical 3 discriminates between the GAG-*gclc* HR and LR. **C** SVM algorithm optimized the difference between patients and controls using the 76 genes but without considering the GAG-*gclc* polymorphism LR/HR genotype, using the 76 genes and the genotype, using the 20 most discriminant genes and the genotype, and finally using the 30 most discriminant genes and the genotype. Accuracy, specificity and sensitivity (with the number of misclassified patients and controls) to discriminate between patients and controls are indicated in the table and in the graph of the ROC curve for the different analysis.

A discriminant analysis on all groups together was performed to find the best split of the data based on four preselected groups of interest. By analyzing all the genes from all groups treated with DMSO or tBHQ, 3 canonical components were sufficient to fully discriminate between the four groups (i.e., LR and HR controls, LR and HR patients; Fig. 5A). The genes that constitute the 3 canonical components are listed in Fig. 5B. The canonical component 1 maximized the discrimination between controls and patients, while the canonical component 3 discriminated the LR vs the HR (Fig. 5A).

Following this promising discriminant capacity, we used a machine-learning approach to test the predictive value of the present set of gene expression data. We used the SVM algorithm to optimize the difference between patients and controls considering the GAG-*gclc* polymorphism. The SVM algorithm applied to all individuals and using the 76 genes, but without assigning the genotype, reached an accuracy of 96% to discriminate between patients and controls, with a sensitivity of 96.6% and a specificity of 93.3% (Fig. 5C), with one patient and two controls being misclassified. When the genotype of each individual was inserted in the algorithm, the accuracy reached 98%, with only one control being misclassified (Fig. 5C). Then, we applied the same algorithm but using the 20 most discriminant genes and the genotype information. This analysis gave the same results as the previous one, giving an accuracy of 98% (Fig. 5C). However, by taking into account the 30 most discriminant genes and the genotype information, the accuracy reached 100%, as all subjects were classified in the right group (Fig. 5C). This highly promising results show that by selecting a mechanism-based set of genes, patients can be discriminated from controls at the early stage of the disease.

## Discussion

In the present study, we aimed at investigating pathways involved in SZ pathology, related or not to the GAG-*gclc* polymorphism, as well as the potential protective mechanism in controls carrying the same genetic risk for redox dysregulation. We also tested the possibility that these pathways may contribute to a genetic signature enabling the identification of patients. We found that the gene expression profile of HR controls was similar to the profile of patients, except for their increased capacity to compensate the GSH system dysregulation by boosting other antioxidant systems such as the TRX/SRX system and the antioxidant defense master regulator Nrf2, which may confer a protection against the pathology. In patients without the GAG-*gclc* genetic risk for redox dysregulation (LR), other risk factors induced a global gene expression dysregulation, affecting especially the antioxidant system. The HR patients failed to regulate their antioxidant system under both basal and pro-oxidant conditions, which may underlie their pathological condition. The discriminant analysis on sub-groups revealed that RAGE and AGMAT were increased in HR patients, suggesting additional risks related to inflammatory and arginine pathways in interaction with this genotype. Finally, our machine-learning approach predicted patients status with an accuracy up to 100%.

The PCA and the correlation matrix completed each other to reveal interesting profiles of patients and controls in interaction with the GAG-*gclc* genetic risk: In the absence of the GAG-*gclc* polymorphism genetic risk for redox dysregulation (LR), the controls and the patients showed a distinct profile under baseline and oxidative challenge conditions. In the PCA, their response to the tBHQ was similar, but their gene expression pattern at basal level was different, leading to a different expression level under stress conditions. This suggests that risk factors different from the GSH deficit genetic risk induce a global gene expression dysregulation in the LR patients at baseline (DMSO). The correlation matrix corroborates this hypothesis, as increased correlation level between various systems was found to be present in LR patients at baseline.

The HR genotype confers a vulnerability to oxidative stress, as the GCL gene expression and activity are reduced (Fig. 1B, C) and GSH level is decreased [9, 11]. Indeed, the PCA showed that both controls and patients with the HR genotype had a similar response to tBHQ and a similar profile of gene expression under DMSO and tBHQ conditions. However, the HR controls increased their regulatory mechanism, as observed in the correlation matrix under DMSO, suggesting that despite their genetic predisposition towards decreased GSH, they are able to regulate other antioxidant defenses. Therefore, the HR controls have a better regulation of the redox system, which protects them from the genetic vulnerability to oxidative stress. Although the HR patients showed similar gene expression profile than the HR controls under baseline and oxidative conditions in the PCA analysis, the correlation matrix did not reveal increased correlations between the different pathways. This lack of regulation may be key in the pathological process in this sub-group of patients. Indeed, the redox dysregulation associated with this genetic predisposition for GSH deficit is not compensated and may contribute to the disease in the HR patients. Interestingly, the difference in profile between LR and HR patients highlights the important role of the GAG-*gclc* polymorphism in stratifying patients.

The PCA analysis highlighted some interesting pathway that were differentially regulated in patients and controls, in interaction with the GAG-*gclc* genetic risk (Supplementary Figs. 6 and 7). Among them, the GSH and the TRX/SRX systems were differentially activated between the LR controls and the other groups. These systems are complementary, and compensate each other [5]. While the Gpx/Gred system reduces ROS using GSH as co-factor, the SRX reduces ROS through the peroxiredoxin (PRX)/TRX system [46]. As the HR show both decreased expression and activity of the key synthesizing enzyme GCL, leading to decreased GSH levels, a compensation with the TRX/SRX system is required to re-establish the redox homeostasis [16]. The PCA also highlighted the role of inflammation, MMPs and collagen formation/degradation. Inflammation is tightly linked to oxidative stress, as ROS can induce pro-inflammatory pathways, while NFkB and Nrf2 interact with each other [28, 47]. This interaction is supported here, as tBHQ stimulation modulates the inflammatory response, and is altered in patients and in the HR controls. Among the inflammatory effect, MMPs can be activated to induce pro-inflammatory mediators, but also extracellular matrix degradation, such as the perineuronal net, which is the specialized extracellular matrix enwrapping PVI, known to play a critical role in sensory perception and cognition [4, 48]. Different MMPs were already suggested to be involved in SZ pathophysiology [49, 50]. Collagen is also linked to MMPs and inflammation, as collagen degradation can be mediated by some MMPs and their degradation products or collagen accumulation can activate inflammatory mediators [31, 51, 52]. Thus, collagen dysregulation, inflammatory markers and MMPs seem to play an important role in the differences between patients and controls, as well as in their response to tBHQ. Interestingly, alteration in the extracellular matrix composition was found in a metabolomic analysis on fibroblasts of EP patients from the same cohort used in our study [12], thus corroborating our results.

As the PCA and the correlation matrix indicated different profiles of all groups, we further investigated the precise mechanisms that may underlie these differences, using a discrimination analysis of sub-groups. This approach allowed us to assess specific questions regarding a protective effect in HR controls or potential pathways that are induced by other risk factors leading to the disease. By comparing the LR controls and the LR patients, we found that antioxidant genes were downregulated in patients, independently of the GAG-*gclc* risk factor. Other pathways, such as inflammation and the MMPs were also found to be related to the difference between patients and controls, which give insight into other risk factors that may lead to these impairments. Interestingly, the comparison of the HR controls and the HR patients stressed the role of RAGE and AGMAT in HR patients. As both controls and patients have the same genetic risk, RAGE and AGMAT increase in patients may be induced by other genetic and/or environmental risk factors, in interaction with the GAG-*gclc* polymorphism that occurred in patients only. In line with these results, we previously found an important role of RAGE shedding by MMP9 in EP patients, as increased RAGE shedding was associated with decreased PFCx GABA level, especially in HR patients [36], reflecting excitatory/inhibitory imbalance. In this context, RAGE seems to be a signature of the sub-groups of patients displaying a genetic risk towards increased oxidative stress. AGMAT is involved in the hydrolyzation of agmatine into putrescine in the arginine degradation cycle. The arginine pathway has been shown to be altered in SZ [53], and more specifically the agmatine, a neurotransmitter and modulator of synaptic transmission, was found to be increased in the blood of SZ patients [54, 55]. Noteworthy, agmatine was shown to block the NMDAR activation [39, 56], linking the NMDAR hypofunction to the arginine pathway. Therefore, our findings suggest a role for AGMAT in relation to the genetic risk for redox dysregulation in EP patients, linking the arginine pathway to the redox balance.

Finally, a machine-learning approach enabled us to investigate the predictive value of our list of genes for a diagnostic purpose. Machine-learning is now an emerging tool in psychiatry [57–59] and was used in several imaging studies to discriminate patients from controls, based on brain structure and connectivity [60–65] or brain activity [66]. In order to highlight potential mechanisms that are involved in the pathophysiology of SZ, many studies have also investigated gene expression profile by microarray or RNAseq analysis on peripheral blood cells or fibroblasts from SZ patients [67–72]. These studies revealed interesting differences in genes belonging to the cell cycle, apoptosis and metabolism, which are the pathways that are predominantly expressed in blood cells and fibroblasts [68, 70, 71]. Noteworthy, a specific profile of gene expression was able to predict the response to antipsychotic treatment in first episode patients, using machine-learning approach, in the same line as our study [72]. In the present study, by choosing some representative genes in selected pathways, we proposed a hypothesis-driven gene expression analysis that allowed to reveal brain related mechanism underlying the differences between patient and controls. Interestingly, the information about the genetic risk for GSH deficit (HR and LR) gave more power to the discrimination by the machine-learning method. More strikingly, a profile of the 30 most discriminant genes could identify patients with an accuracy of 100%, which is unique, to our knowledge. Still, the major limitation of our study is the small sample size (*N* = 30). Therefore, this approach needs further validation with an independent and larger cohort in order to generalize this approach for the development of a personalized output to enhance the prediction accuracy of clinical measures and to develop early intervention strategies.

In conclusion, our computational approach based on the expression of genes related to hypothesis-driven pathways highlighted some mechanisms involved in the early pathophysiology of SZ. We found specific signatures converging on oxidative stress even in patients not carrying the GAG-*gclc* genetic risk for redox dysregulation. In contrast, we identified compensatory antioxidant mechanisms that protect the controls bearing the same genetic risk. Moreover, *agmat* and *rage* gene expressions were involved only in HR patients, revealing its interaction with other risk factors such as inflammation. Finally, we could predict the SZ status with an accuracy up to 100%. Thus, by combining machine learning with a well-chosen set of genes, we identified novel disease-related pathways and obtained a highly-accurate approach to identify patients at the early stage of the disease. In turn, this approach may improve early detection and intervention for the disease.

### Supplementary information

Supplementary Material and Method

Supplementary Figure 1

Supplementary Figure 2

Supplementary Figure 3

Supplementary Figure 4

Supplementary Figure 5

Supplementary Figure 6

Supplementary Figure 7

Supplementary Table 1

Supplementary Table 2

Supplementary Table 3
