# Supplementary material for: Characterization of early psychosis patients carrying a genetic vulnerability to redox dysregulation: a computational analysis of mechanism-based gene expression profile in fibroblasts
Source: Mol Psychiatry. 2023 Mar 31;28(5):1983–94. doi: 10.1038/s41380-023-02034-x (PMC10575782; doi:10.1038/s41380-023-02034-x)
Supplement: Supplementary file 1 — Supplementary Material and Method [file 41380_2023_2034_MOESM1_ESM.docx]

**Supplementary Materials and Methods**

Subjects recruitment

EP patients were recruited from the Treatment and Early Intervention in Psychosis Program (TIPP), a 3-year specialized program for patients aged between 15 and 35 years old who met the threshold criteria for psychosis according to the Comprehensive Assessment of At-Risk Mental States criteria). [20, 21].

Symptom severity at the time of biopsy was assessed using the Positive And Negative Syndrome Scale (PANSS)[73] by trained psychologists. Healthy controls were recruited and assessed by the Diagnostic Interview for Genetic Studies[74], with the following exclusion criteria: Major mood, psychotic or substance-use disorder and having a ﬁrst-degree relative with a psychotic disorder. All subjects were enrolled following a fully informed written consent. This work was carried out in accordance with the Declaration of Helsinki and was approved by the local Ethics Committee (*Commission cantonale d’éthique de la recherche sur l’être humain (CER-VD)*). Patients and controls were age-matched and no statistical differences were found for sex, ethnicity and BMI. No differences within the patient groups were found for illness severity (PANSS), illness duration and medication (Suppl Table 2).

Subjects genotyping

The GAG trinucleotide-repeat polymorphism in *the gclc* gene was genotyped as previously described [74] and assigned to the GAG-*gclc* high-risk or GAG-*gclc* low-risk genotype based on the number of GAG repeats, as defined in Gysin et al. As discussed previously [9, 75], no SNP haplotype has been found to be a perfect surrogate for any of the GAG repeat length in *gclc* gene. Therefore, they cannot be derived from available SNP data from genome-wide association studies (GWAS) and a standard methodology should be used to correctly genotype these alleles[75].

Machine learning Analysis

A machine-learning algorithm Support Vector Machine (SVM) was performed in order to optimize the difference between patients and controls considering the HR/LR genotype. This algorithm finds out the best hyperplanes in high-dimensional space, maximizing the gap between each point, in order to find the best separation between the groups. The process tests its predictive capacity to classify each data point in the right category using the Leave-One-Out method, which consists of a training set of P-1 individuals and using the one left to test the model. This training/testing is repeated P times, and the model adjusts itself in a linear manner to obtain the best prediction.

**Supplementary Figure and Table Legends**

**Suppl Figure 1:** Boxplot of genes from (A) the GSH, (B) the antioxidant and (C) the arginine pathways, after a 2 way-ANOVA analysis with 3 factors, the treatment (T: tBHQ and DMSO), the status (Gr: patient or control) and the genotype (G: GAG-*gclc* HR and LR). Data are expressed as mean ± s.e.d. (N=15) * P <0.05; ** P <0.01; *** P <0.001.

**Suppl Figure 2:** Boxplot of genes from (A) the inflammation and (B) the complement pathways, after a 2 way-ANOVA analysis with 3 factors, the treatment (T: tBHQ and DMSO), the status (Gr: patient or control) and the genotype (G: GAG-*gclc* HR and LR). Data are expressed as mean ± s.e.d. (N=15) * P <0.05; ** P <0.01; *** P <0.001.

**Suppl Figure 3:** Boxplot of genes from (A) the MMPs and (B) RAGE pathways, after a 2 way-ANOVA analysis with 3 factors, the treatment (T: tBHQ and DMSO), the status (Gr: patient or control) and the genotype (G: GAG-*gclc* HR and LR). Data are expressed as mean ± s.e.d. (N=15) * P <0.05; ** P <0.01; *** P <0.001.

**Suppl Figure 4:** Boxplot of genes from (A) the GABA, (B) BDNF and (C) the collagen pathways, after a 2 way-ANOVA analysis with 3 factors, the treatment (T: tBHQ and DMSO), the status (Gr: patient or control) and the genotype (G: GAG-*gclc* HR and LR). Data are expressed as mean ± s.e.d. (N=15) * P <0.05; ** P <0.01; *** P <0.001.

**Suppl Figure 5:** PCA analysis. (A) Representation of all subjects visualized based on PC1 and PC2, showing their contribution of 27.2% and 12.2% to the variability, respectively, and the graph for the eigenvalues of the other components.

**Suppl Figure 6:** Representation of the factorial analysis of rotated PCA with a focus on the pathways. (A) Graphic representation of the different pathways that contribute to factor1 and factor2. Factor1 explained the response to tBHQ in the X-axis and factor2 separated the 4 groups in the Y-axis. (B) Highlight of each gene indicated in the PCA under its pathway to identify its contribution to the factor1 and factor2. Pathways represented are GSH, antioxidants (AO), inflammation and MMPs.

**Suppl Figure 7:** Representation of the factorial analysis of rotated PCA with a focus on the pathways. Highlight of each gene indicated in the PCA under its pathway to identify its contribution to the factor1 and factor2. Pathways represented are RAGE, GABA, collagen, arginine, BDNF and complement.

**Suppl Table 1:** Table of the genes that were chosen for expression analysis in fibroblasts of EP patients and age matched controls, with the GAG-*gclc* polymorphism. Gene name is indicated, as well as the protein name, the full name and the pathway to which it belongs.

**Suppl Table 2:** Demographic table of EP patients and controls, with the GAG-*gclc* polymorphism that were involved in the study for skin-biopsy derived fibroblasts. Note: CPZ equivalents, chlorpromazine equivalents; p-value for 2-way ANOVA (group and genotype effect) or t-test (for illness duration and CPZ equivalent).

**Suppl Table 3:** Total of genes that contribute to factor1 and factor2 in the rotated PC.
