# Supplementary figures and images for: Characterization of early psychosis patients carrying a genetic vulnerability to redox dysregulation: a computational analysis of mechanism-based gene expression profile in fibroblasts
Source: Mol Psychiatry. 2023 Mar 31;28(5):1983–94. doi: 10.1038/s41380-023-02034-x (PMC10575782; doi:10.1038/s41380-023-02034-x)

### A. GSH

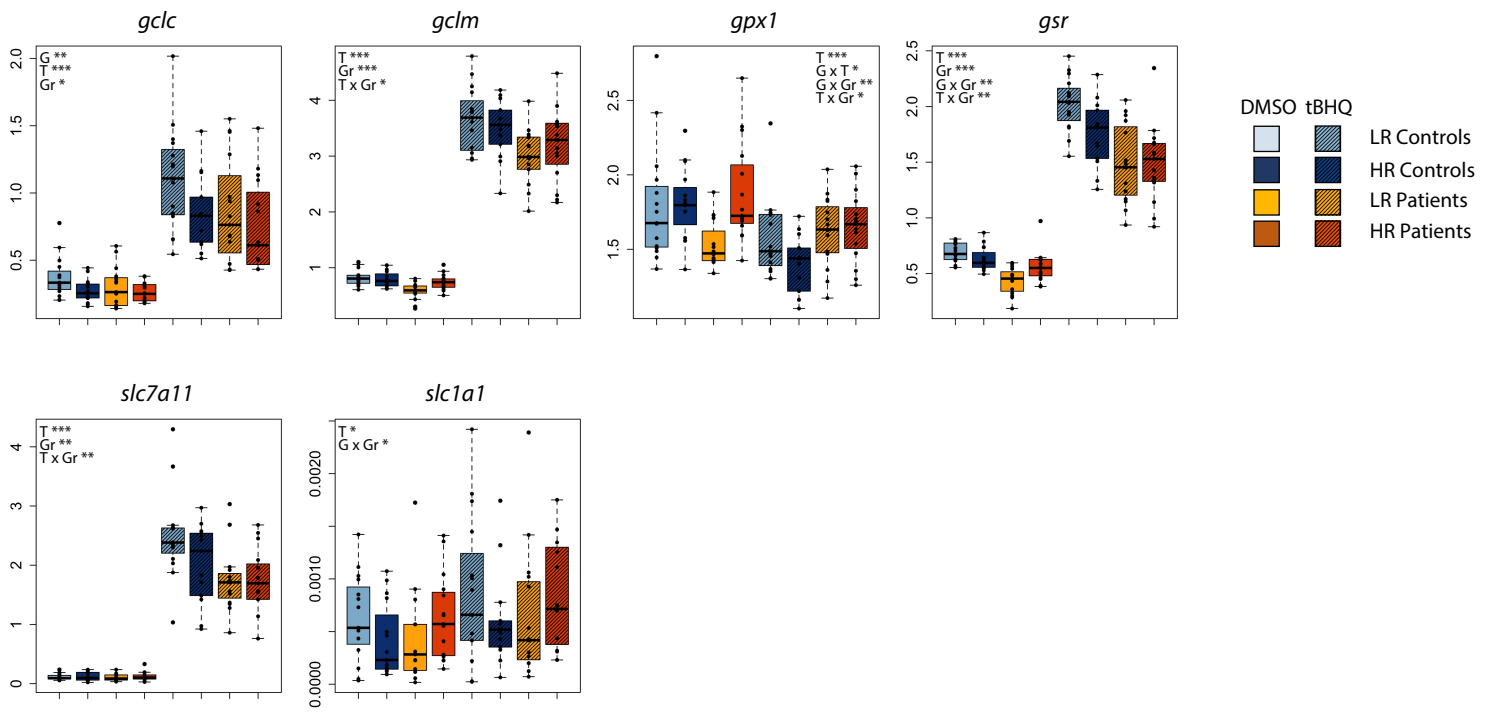

## B. Antioxidant

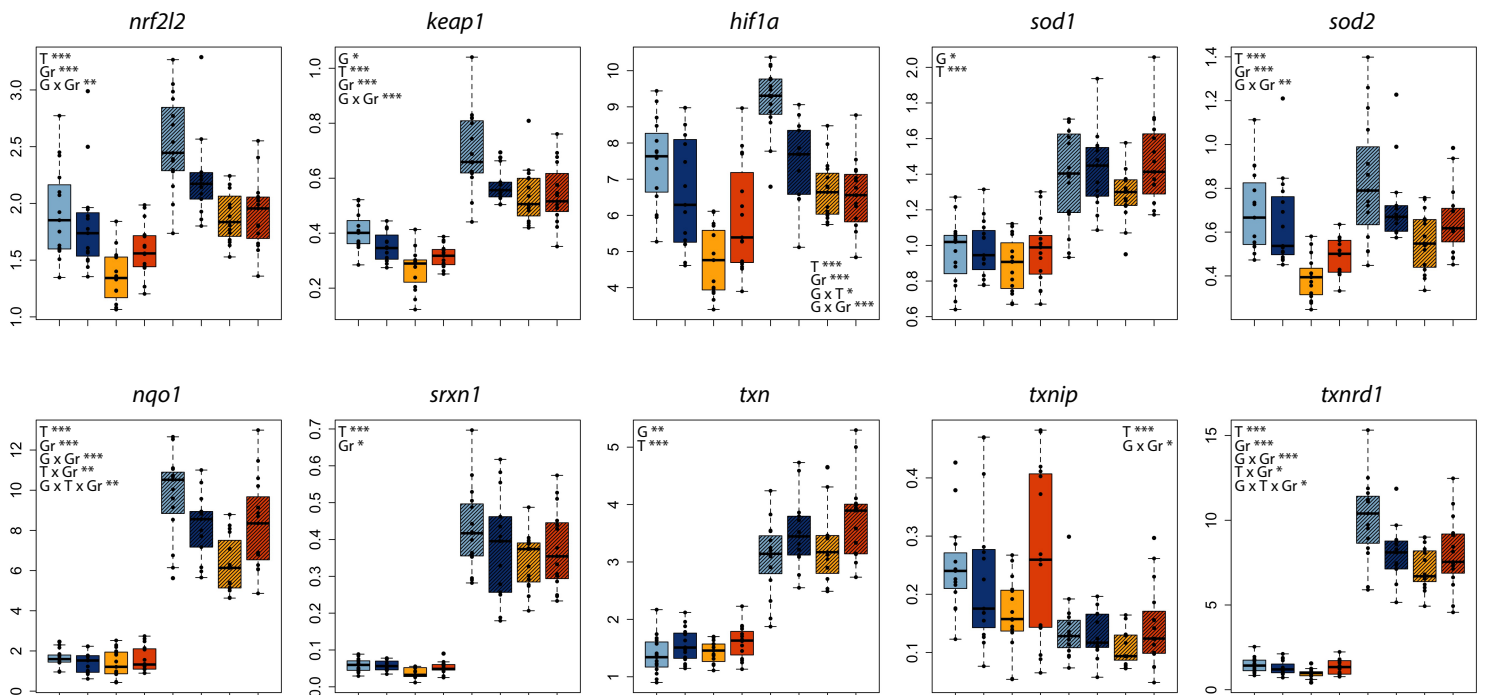

### C. Arginin

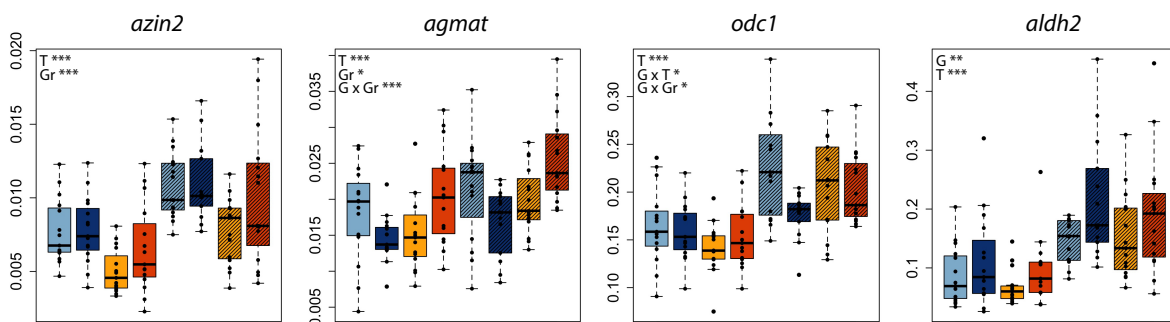

Supplement: Supplementary file 2 — Supplementary Figure 1 [file 41380_2023_2034_MOESM2_ESM.pdf]

A. Inflammation

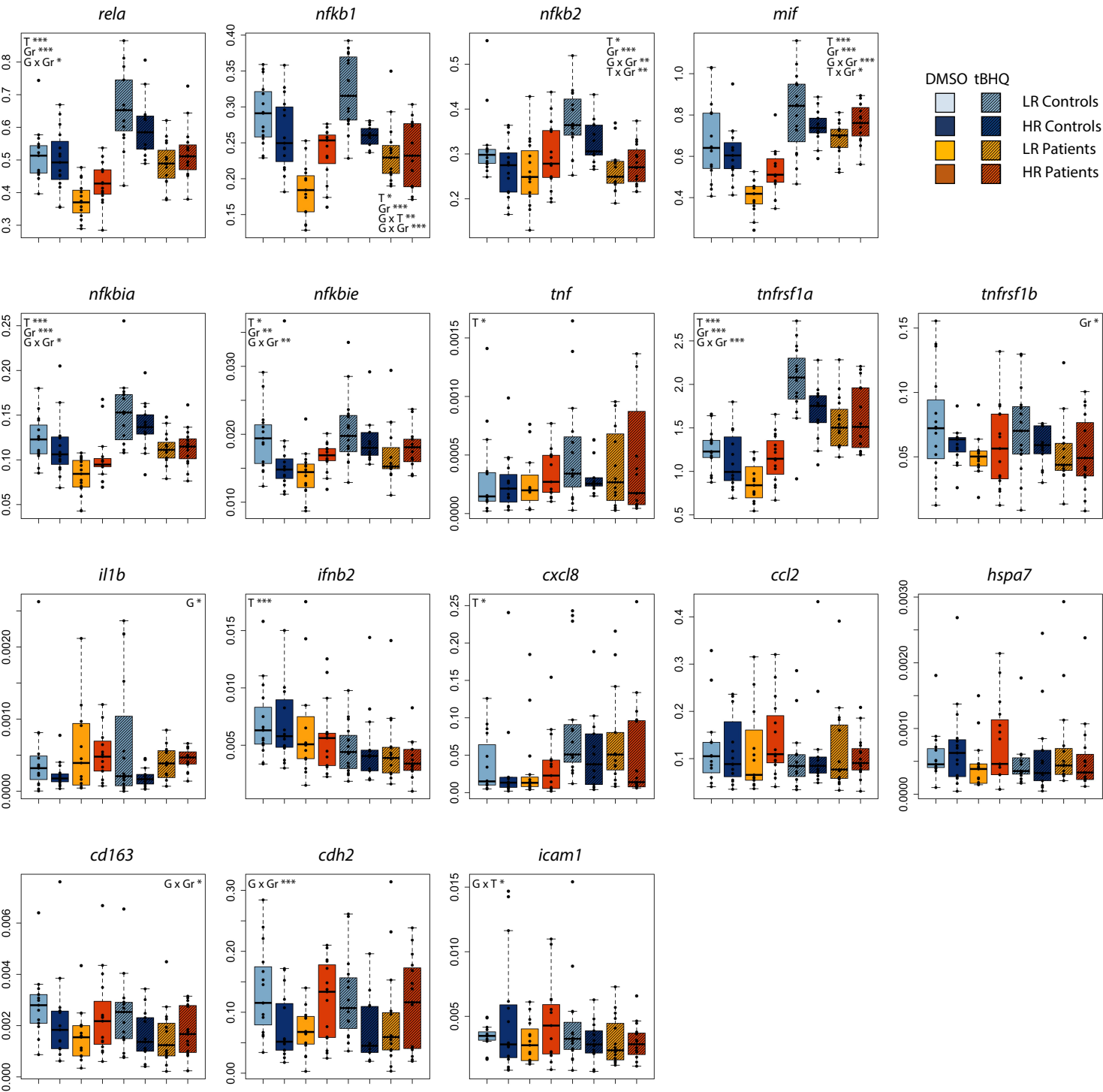

B. Complement

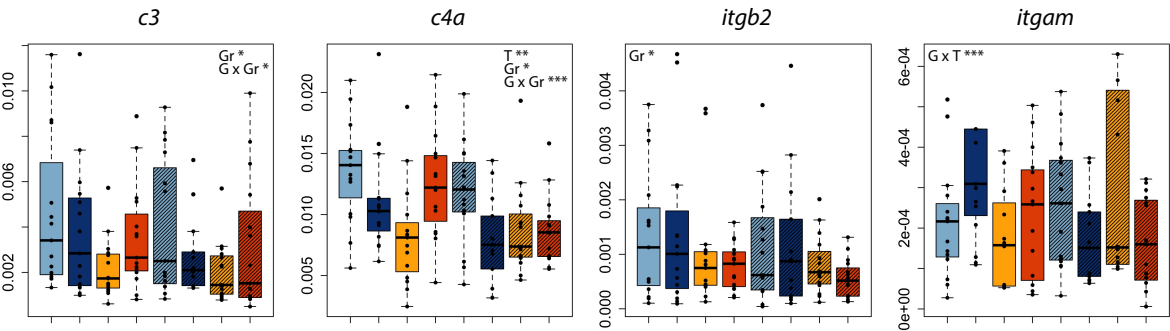

Supplement: Supplementary file 3 — Supplementary Figure 2 [file 41380_2023_2034_MOESM3_ESM.pdf]

A. MMPs

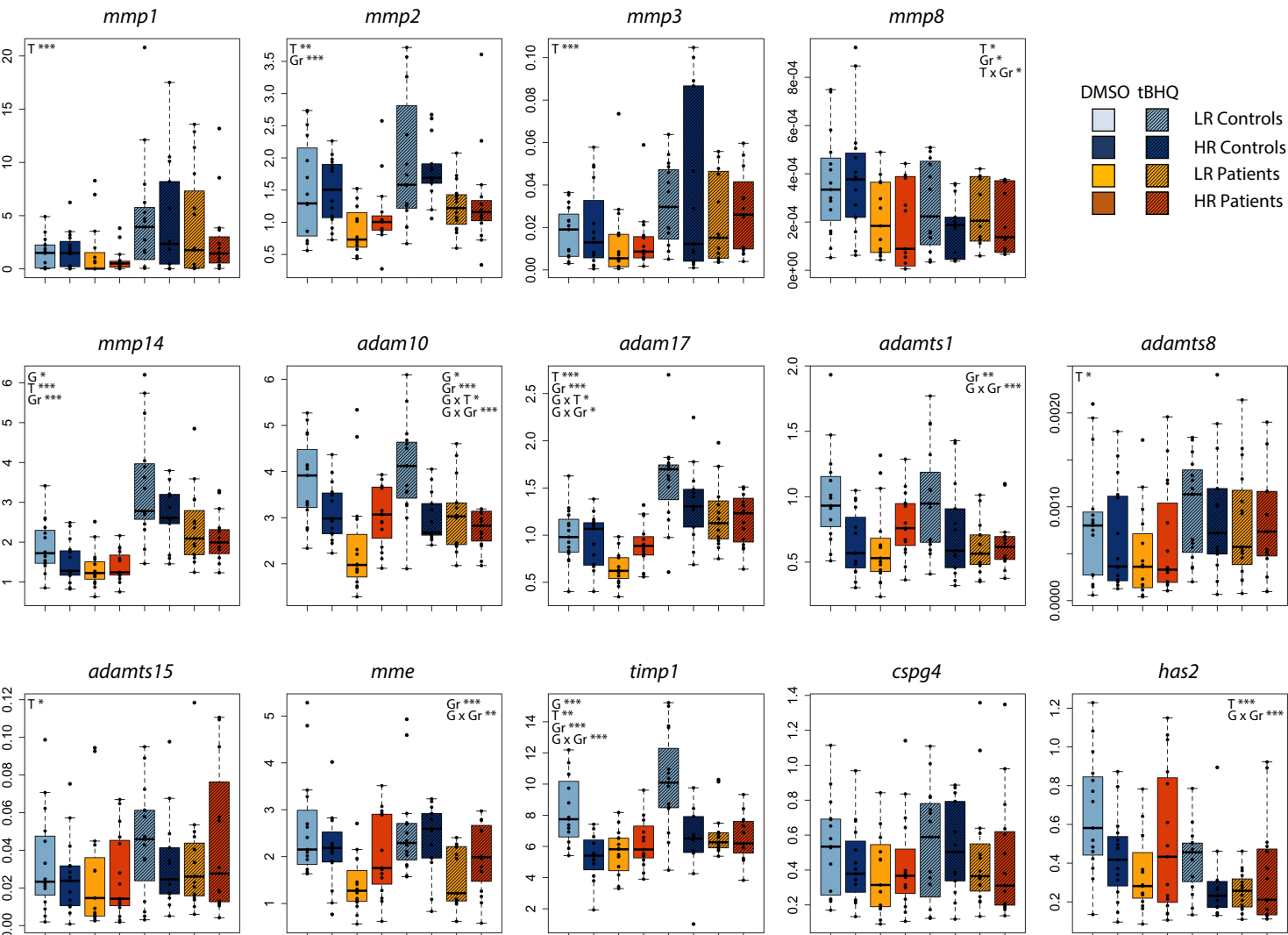

B. RAGE

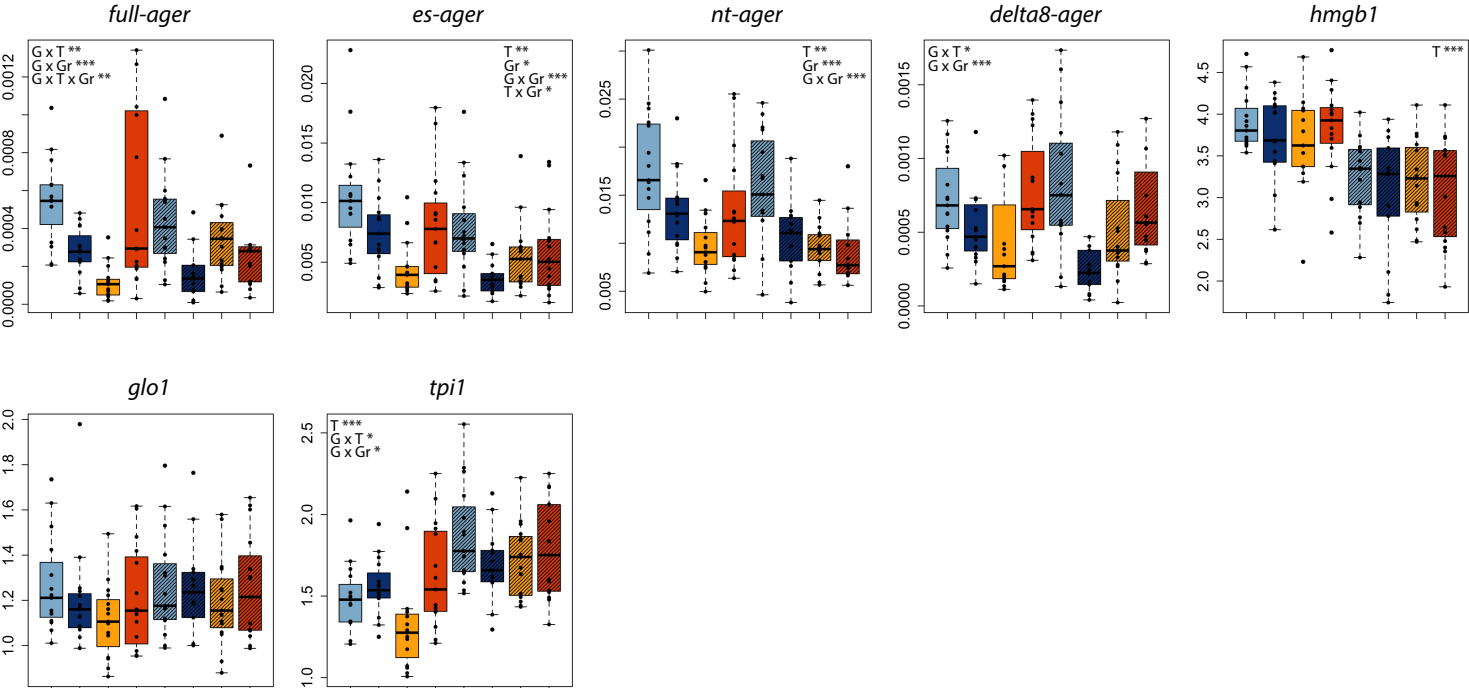

Supplement: Supplementary file 4 — Supplementary Figure 3 [file 41380_2023_2034_MOESM4_ESM.pdf]

A. GABA

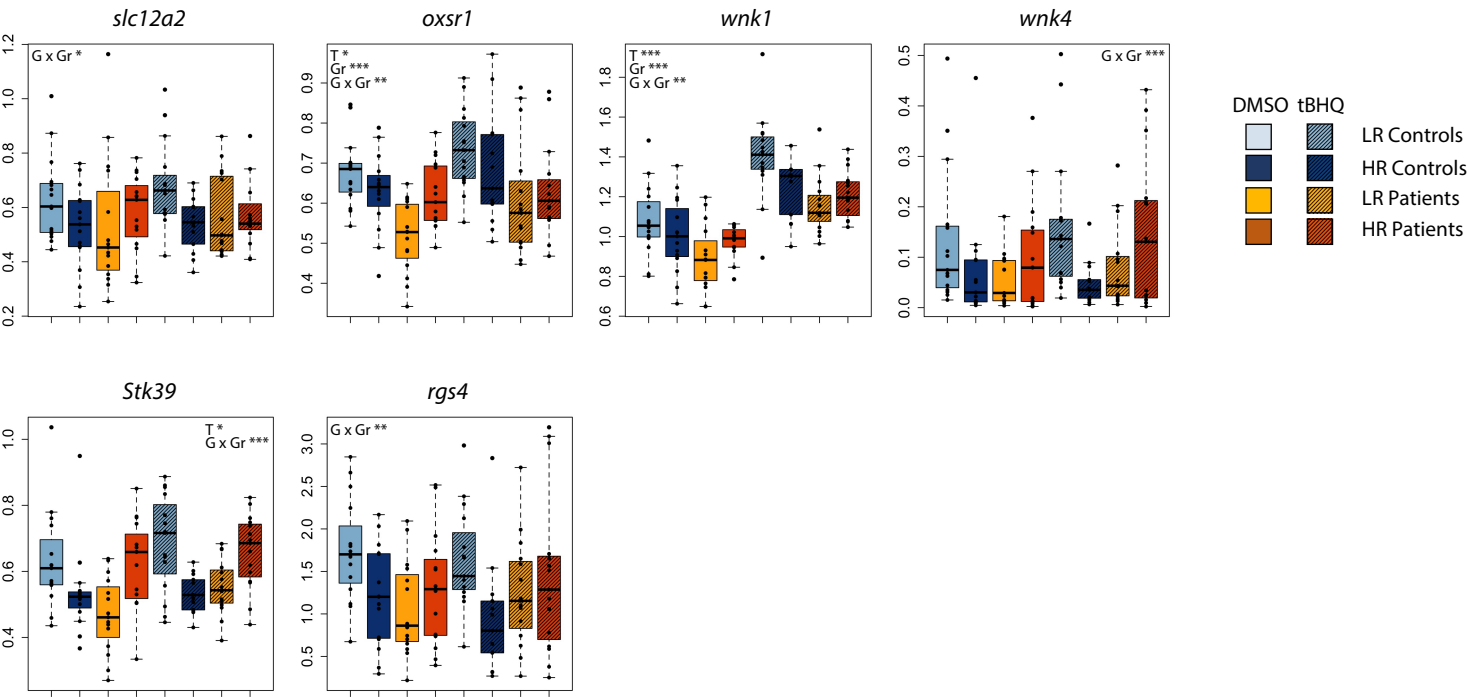

B. BDNF

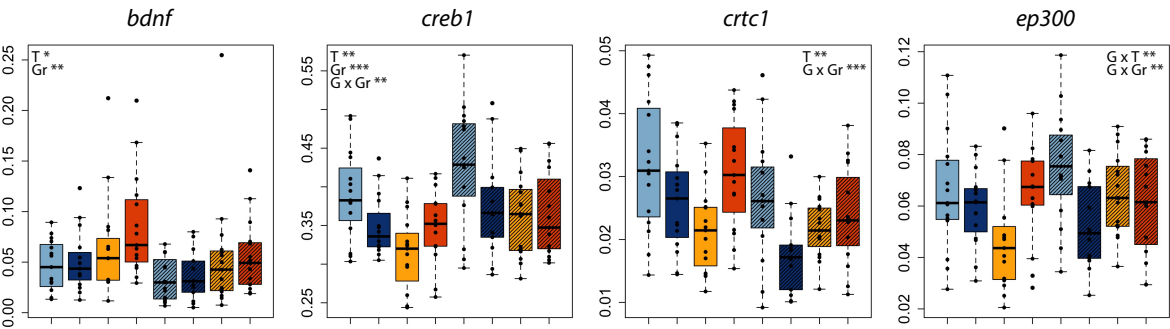

C. Collagen

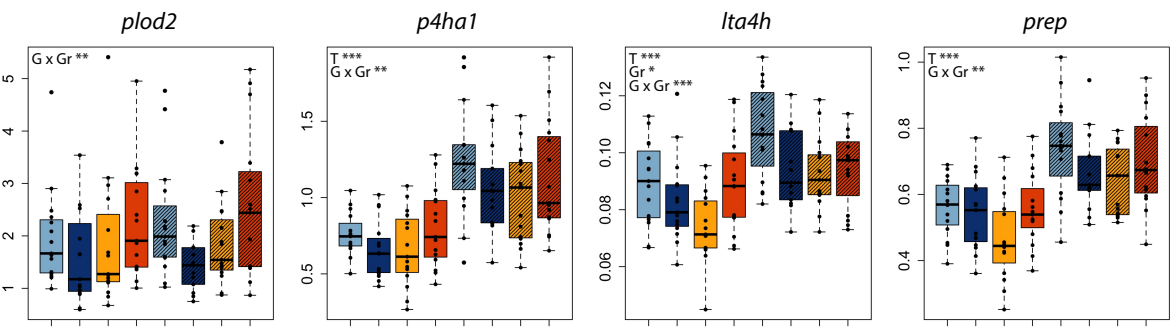

Supplement: Supplementary file 5 — Supplementary Figure 4 [file 41380_2023_2034_MOESM5_ESM.pdf]

A.

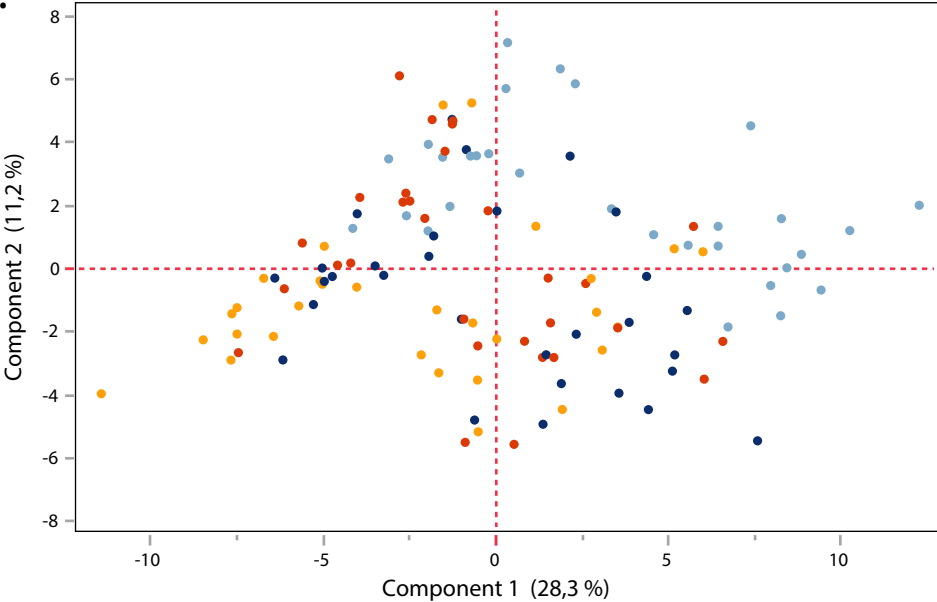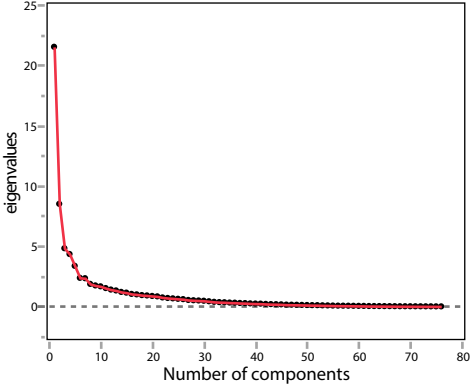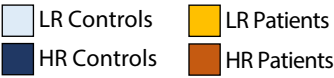

Supplement: Supplementary file 6 — Supplementary Figure 5 [file 41380_2023_2034_MOESM6_ESM.pdf]

A.

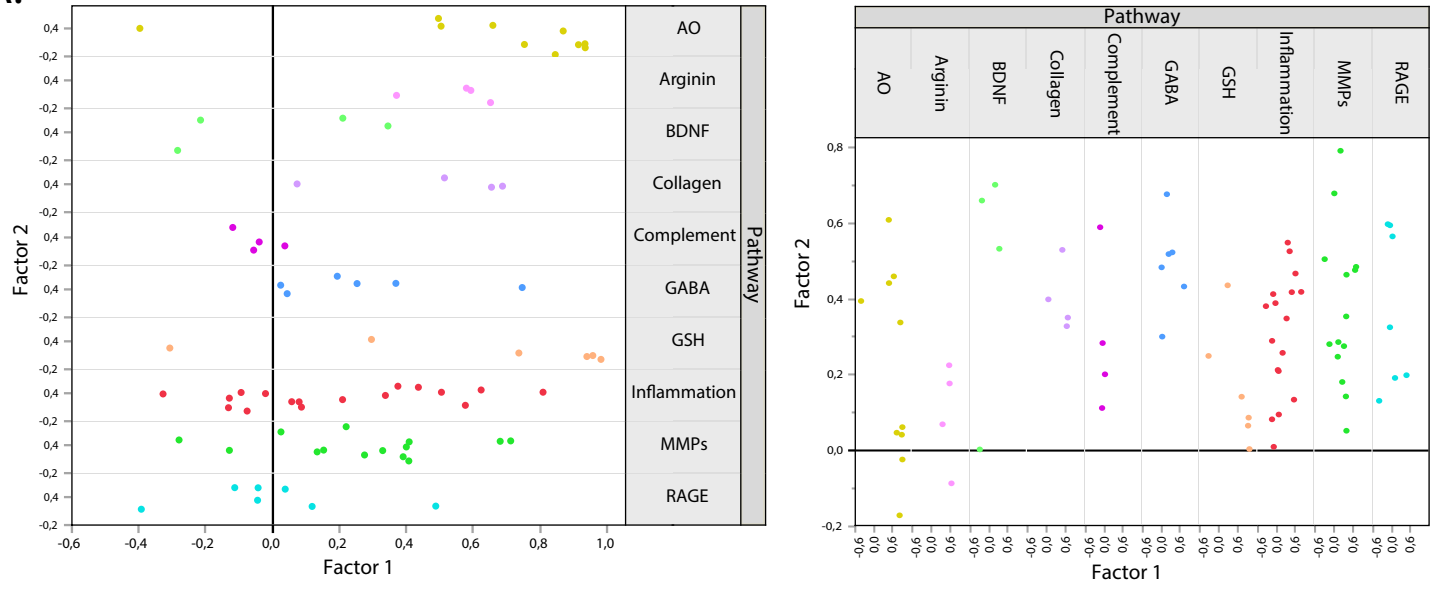

B.

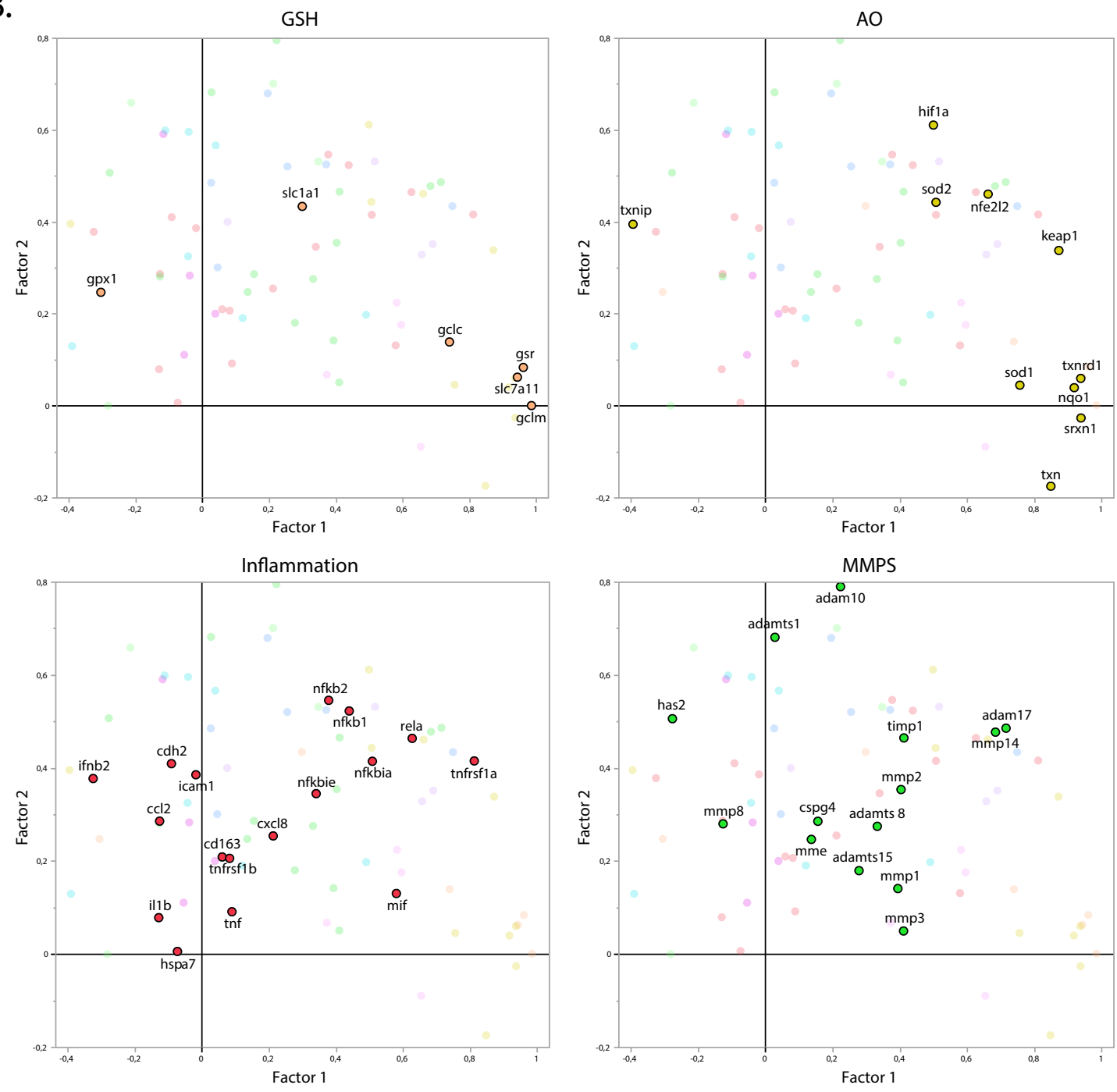

Supplement: Supplementary file 7 — Supplementary Figure 6 [file 41380_2023_2034_MOESM7_ESM.pdf]

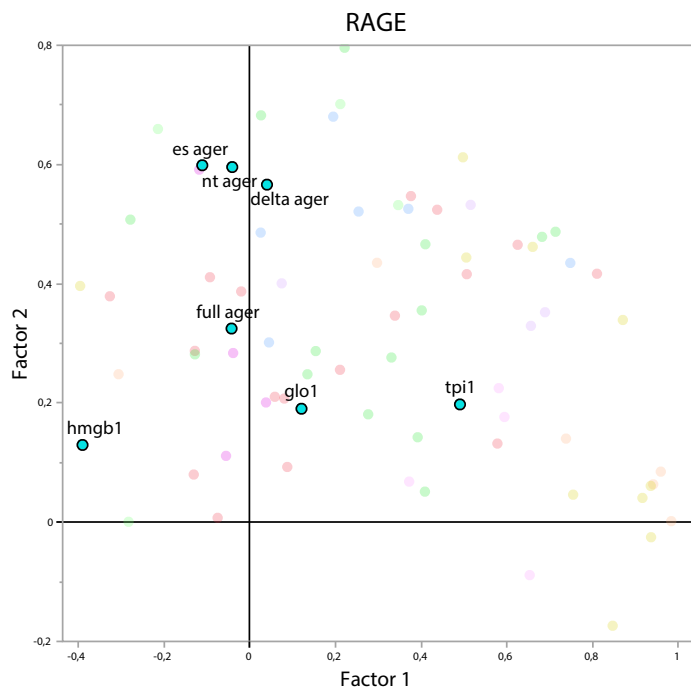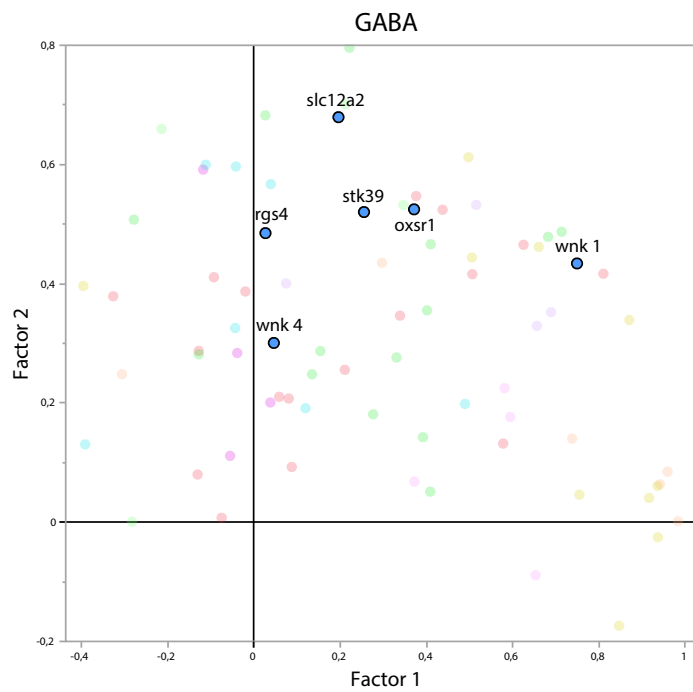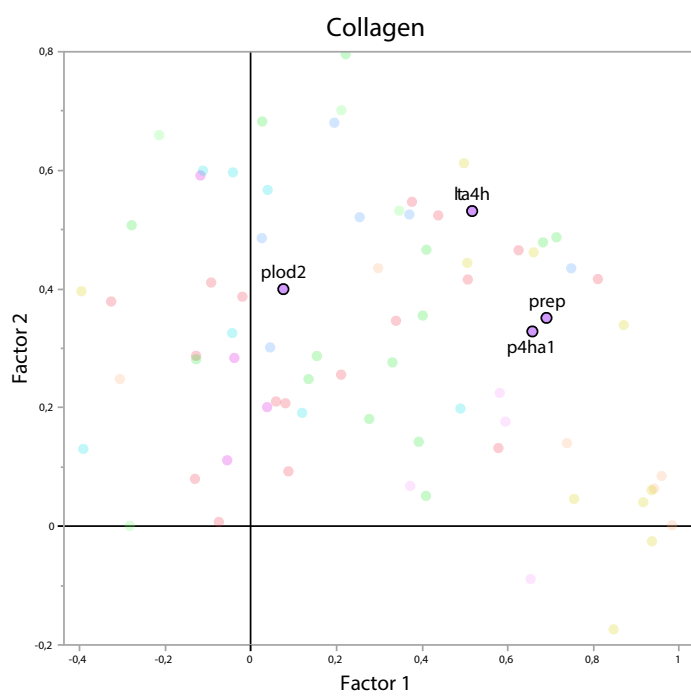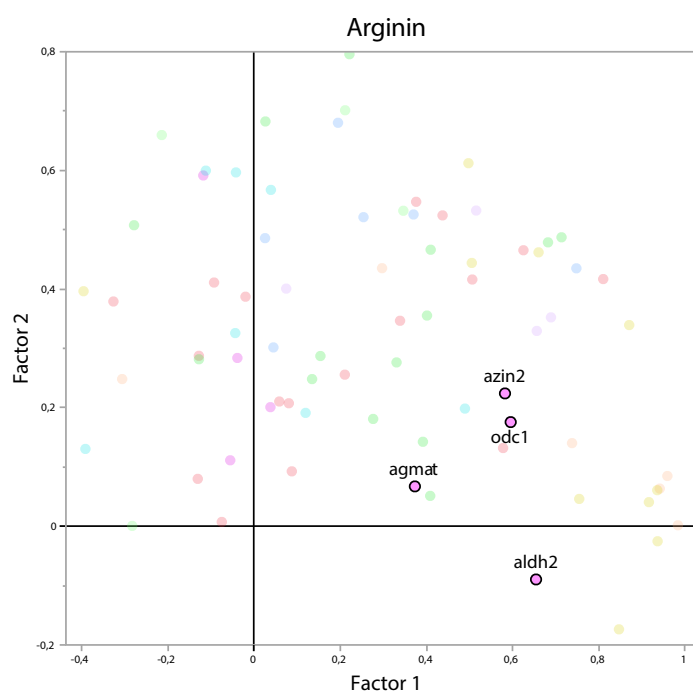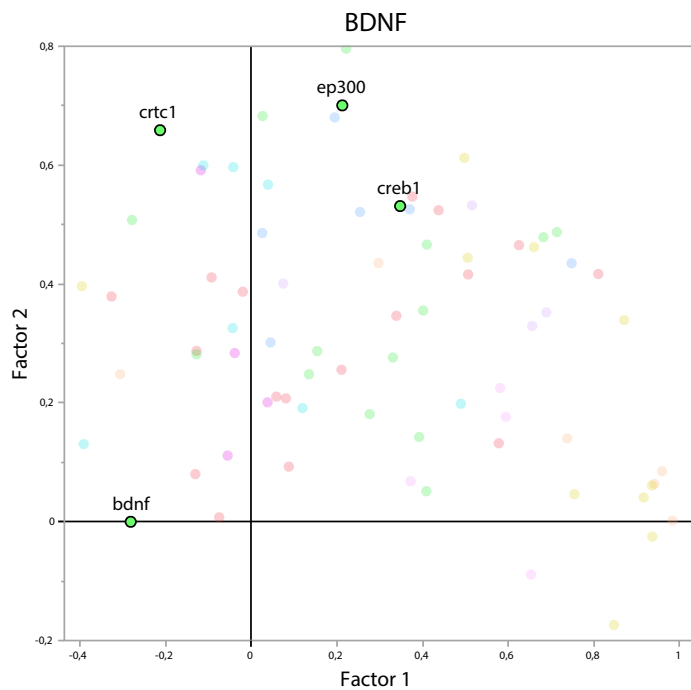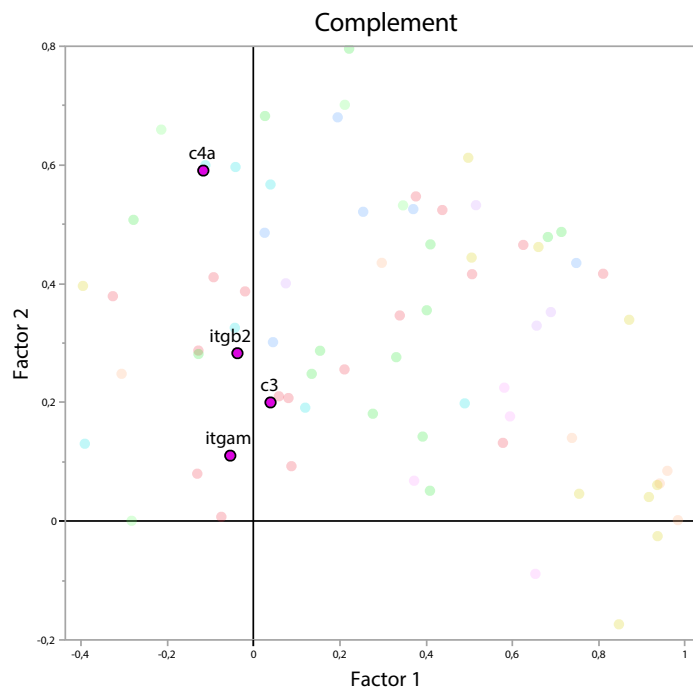

Supplement: Supplementary file 8 — Supplementary Figure 7 [file 41380_2023_2034_MOESM8_ESM.pdf]
