# Supplementary material for: Characterization of early psychosis patients carrying a genetic vulnerability to redox dysregulation: a computational analysis of mechanism-based gene expression profile in fibroblasts
Source: Mol Psychiatry. 2023 Mar 31;28(5):1983–94. doi: 10.1038/s41380-023-02034-x (PMC10575782; doi:10.1038/s41380-023-02034-x)
Supplement: Supplementary file 9 — Supplementary Table 1 [file 41380_2023_2034_MOESM9_ESM.docx]

| **Pathway** | **Specific pathway** | **Gene** | **Protein** | **Full name** |
| --- | --- | --- | --- | --- |
| Redox | GSH system | *slc7a11* | xCT | Solute Carrier Family 7 |
| Redox | GSH system | *gclm* | GCLM | Glutamate-cysteine ligase, modifier subunit |
| Redox | GSH system | *gclc* | GCLC | Glutamate-cysteine ligase, catalytic subunit |
| Redox | GSH system | *gsr* | GR | Glutathione reductase |
| Redox | GSH system | *gpx1* | GPX1 | Glutathione peroxidase 1 |
| Redox | GSH system | *slc1a1* | EAAT3 | Solute carrier family 1 member 1 |
| Redox | antioxidant | *nfe2l2* | Nrf2 | Nuclear factor erythroid 2-related factor 2 |
| Redox | antioxidant | *keap1* | KEAP1 | Kelch-like ECH-associated protein 1 |
| Redox | antioxidant | *hif1a* | HIF-1alpha | Hypoxia-inducible factor |
| Redox | antioxidant | *sod1* | SOD1 | Superoxide dismutase 1, |
| Redox | antioxidant | *sod2* | SOD2 | Manganese Superoxide dismutase |
| Redox | antioxidant | *nqo1* | NQO1 | NAD(P)H dehydrogenase, quinone 1 |
| Redox | TRX/SRX | *txnrd1* | TXNRD1 | Thioredoxin reductase 1 |
| Redox | TRX/SRX | *txnip* | Txnip | Thioredoxin interacting protein |
| Redox | TRX/SRX | *txn* | TRX | Thioredoxin |
| Redox | TRX/SRX | *srxn1* | SRX | Sulfiredoxin |
| ECM / Inflammation | MMPs | *adam10* | ADAM10 | ADAM metallopeptidase domain 10 |
| ECM / Inflammation | MMPs | *adam17* | ADAM17 | ADAM metallopeptidase domain 17 |
| ECM / Inflammation | MMPs | *mmp14* | MMP1 | Matrix metalloproteinase 1 |
| ECM / Inflammation | MMPs | *mmp14* | MMP14 | Matrix metalloproteinase 14 |
| ECM / Inflammation | MMPs | *mmp2* | MMP2 | Matrix metalloproteinase 2 |
| ECM / Inflammation | MMPs | *mmp3* | MMP3 | Matrix metalloproteinase 3 |
| ECM / Inflammation | MMPs | *mmp8* | MMP8 | Matrix metalloproteinase 8 |
| ECM / Inflammation | MMPs | *timp1* | TIMP1 | tissue inhibitor of metalloproteinases |
| ECM / Inflammation | MMPs | *mme* | Neprilysin | Membrane metallo-endopeptidase |
| ECM / Inflammation | MMPs | *adamts15* | ADAMTS1 | ADAM metallopeptidase with thrombospondin type 1 motif, 1 |
| ECM / Inflammation | MMPs | *adamts8* | ADAMTS 8 | ADAM metallopeptidase with thrombospondin type 1 motif, 8 |
| ECM / Inflammation | MMPs | *adamts15* | ADAMTS15 | ADAM metallopeptidase with thrombospondin type 1 motif, 15 |
| ECM / Inflammation | ECM | *cspg4* | Cspg4 | Chondroitin sulfate proteoglycan 4 |
| ECM / Inflammation | ECM | *has2* | HAS2 | Hyaluronan synthase 2 |
| Inflammation | inflammation | *rela* | p65 | Nuclear factor-kappa B p65 |
| Inflammation | inflammation | *nfkb1* | p105 | Nuclear factor-kappa B p105 |
| Inflammation | inflammation | *nfkb2* | p100/p52 | Nuclear factor-kappa B p52 |
| Inflammation | inflammation | *cxcl8* | IL-8 | Interleukine 8 |
| Inflammation | inflammation | *ifnb2* | IL-6 | Interleukine 6 |
| Inflammation | inflammation | *nfkbia* | IkB-a | NF-kB inhibitor alpha |
| Inflammation | inflammation | *nfkbie* | IkB-e | NF-kB inhibitor epsilon |
| Inflammation | inflammation | *mif* | MIF | Macrophage migration inhibitory factor |
| Inflammation | inflammation | *hspa1a* | Hsp70 | Heat shock 70 kDa protein 1A |
| Inflammation | inflammation | *tnf* | TNFa | Tumor necrosis factor alpha |
| Inflammation | inflammation | *tnfrsf1a* | TNFR1/p55 | TNFa receptor p55 |
| Inflammation | inflammation | *tnfrsf1b* | TNFR2/p75 | TNFa receptor p75 |
| Inflammation | inflammation | *il1b* | IL-1b | Interleukine 1B |
| Inflammation | inflammation | *cd163* | CD163 | Scavenger receptor cysteine-rich type 1 protein M130 |
| Inflammation | cell adhesion | *cdh2* | N-Cadherin | Cadherin-2 |
| Inflammation | cell adhesion | *icam1* | ICAM1 | Intercellular adhesion molecule 1 |
| Inflammation | cell adhesion | *ccl2* | CCL2 | Chemokine (C-C motif) ligand 2 |
| Inflammation | Collagene | *prep* | PE | Prolyl endopeptidase |
| Inflammation | Collagene | *lta4h* | LTA4H | Leukotriene A4 hydrolase |
| Inflammation | Collagene | *p4ha1* | P4HA1 | Prolyl 4-hydroxylase subunit alpha-1 |
| Inflammation | Collagene | *plod2* | PLOD2 | Procollagen-Lysine, 2-Oxoglutarate 5-Dioxygenase 2 |
| Inflammation | Complement | *c4a* | C4A | Complement 4 |
| Inflammation | Complement | *c3* | C3 | Complement 3 |
| Inflammation | Complement | *itgam* | CD11b | part of Complement receptor 3 (C3R) |
| Inflammation | Complement | *itgb2* | CD18 | part of Complement receptor 3 (C3R) |
| NMDAR / Redox | Arginine | *odc1* | ODC1 | Ornithine decarboxylase |
| NMDAR / Redox | Arginine | *azin2* | ADC | Arginine decarboxylase |
| NMDAR / Redox | Arginine | *aldh2* | aldDH | Aldehyde dehydrogenase 2 family |
| NMDAR / Redox | Arginine | *agmat* | AGMAT | Agmatinase |
| RAGE | RAGE | *full ager* | RAGE | Receptor for advanced glycation end-product Full-lenght |
| RAGE | RAGE | *es ager* | esRAGE | Receptor for advanced glycation end-product endogenous secreted |
| RAGE | RAGE | *nt ager* | Nt-RAGE | Receptor for advanced glycation end-product N-terminal truncated |
| RAGE | RAGE | *delta ager* | Delta-RAGE | Receptor for advanced glycation end-product Delta8 |
| RAGE | RAGE | *hmgb1* | HMGb1 | High Mobility Group Box 1 |
| RAGE | AGE | *glo1* | GLO1 | Glyoxalase I |
| RAGE | AGE | *tpi1* | TPI1 | Triosephosphate isomerase 1 |
| GABAergic development | NKCC1/KCC2 | *slc12a2* | NKCC1 | Solute carrier family 12 (sodium/potassium/chloride transporter), member 2 |
| GABAergic development | NKCC1/KCC2 | *oxsr1* | OSR1 | Serine/threonine-protein kinase OSR1 |
| GABAergic development | NKCC1/KCC2 | *rgs4* | Rgs4 | Regulator of G-protein signaling 4 |
| GABAergic development | NKCC1/KCC2 | *wnk1* | WNK 1 | Serine/threonine-protein kinase WNK1 |
| GABAergic development | NKCC1/KCC2 | *wnk4* | WNK 4 | Serine/threonine-protein kinase WNK4 |
| GABAergic development | NKCC1/KCC2 | *stk39* | SPAK | STE20/SPS1-related proline-alanine-rich protein kinase |
| Brain maturation | BDNF | *bdnf* | BDNF | Brain-derived neurotrophic factor |
| Brain maturation | BDNF | *creb1* | CREB | CAMP Responsive Element Binding Protein 1 |
| Brain maturation | BDNF | *ep300* | P300 | E1A Binding Protein P300 |
| Brain maturation | BDNF | *crtc1* | CRTC1 | CREB Regulated Transcription Coactivator 1 |
| Housekeeping |  | *rps26* | RS26 | Ribosomal Protein S26 |
| Housekeeping |  | *cdkn1b* | CDKN1B | Cyclin-Dependent Kinase Inhibitor 1B |
| Housekeeping |  | *hprt1* | HPRT1 | Hypoxanthine Phosphoribosyltransferase 1 |
| Housekeeping |  | *tubb* | Tubb | Tubulin beta class I |
| Housekeeping |  | *rpl27* | RPL27 | Ribosomal Protein L27 |
| Housekeeping |  | *36b4* | RPLP0 | Acidic ribosomal phosphoprotein P0 |
