# Supplementary material for: Characterization of early psychosis patients carrying a genetic vulnerability to redox dysregulation: a computational analysis of mechanism-based gene expression profile in fibroblasts
Source: Mol Psychiatry. 2023 Mar 31;28(5):1983–94. doi: 10.1038/s41380-023-02034-x (PMC10575782; doi:10.1038/s41380-023-02034-x)
Supplement: Supplementary file 10 — Supplementary Table 2 [file 41380_2023_2034_MOESM10_ESM.docx]

|  |  | **Control GAG-*gclc* LR (N=15)** | | **Control GAG-*gclc* HR (N=15)** | | **Patient GAG-*gclc* LR (N=15)** | | **Patient GAG-*gclc* HR (N=15)** | | **p value** |
| --- | --- | --- | --- | --- | --- | --- | --- | --- | --- | --- |
|  |  |  |  |  |  |  |  |  |  |  |
|  |  |  |  |  |  |  |  |  |  |  |
|  |  |  |  |  |  |  |  |  |  |  |
| **Age (years, mean±s.d.)** | | 26.8 ± 5.1 | | 26.3 ± 46.5 | | 25.6 ± 6.6 | | 25.7 ± 5.8 | | 0.85 |
| **BMI (kg/m2, means±s.d.)** | | 23.13 ± 1.9 | | 24.1 ± 1.2 | | 23.7 ± 3.4 | | 24.5 ± 3.1 | | 0.07 |
| **Illness duration (years, mean±s.d.)** | | NA | | | | 2.5 ± 1 | | 1.9 ± 1.3 | | 0.28 |
| **CPZ equivalent (mg, mean±s.d.)** | | NA | | | | 390.1 ± 230.8 | | 213.9 ± 171.7 | | 0.068 |
|  |  |  |  |  |  |  |  |  |  |  |
